# The medical competence of health care providers in sub-Saharan Africa: Evidence from 16 127 providers across 11 countries

**DOI:** 10.1093/haschl/qxae066

**Published:** 2024-06-07

**Authors:** Benjamin Daniels, Andres Yi Chang, Roberta Gatti, Jishnu Das

**Affiliations:** McCourt School of Public Policy, Georgetown University, Washington, DC 20057, United States; Office of the Chief Economist for Human Development, The World Bank, Washington, DC 20433, United States; Office of the Chief Economist for Middle East and North Africa, The World Bank, Washington, DC 20433, United States; McCourt School of Public Policy and the Walsh School of Foreign Service, Georgetown University, Washington, DC 20057, United States; Centre for Policy Research, New Delhi 110021, India

**Keywords:** health care providers, quality of care, medical vignettes, sub-Saharan Africa, health systems, service delivery indicators, SDI

## Abstract

Despite a consensus that quality of care is critically deficient in low-income countries, few nationally representative studies provide comparable measures of quality of care across countries. To address this gap, we used nationally representative data from in-person administrations of clinical vignettes to measure the competence of 16 127 health care providers across 11 sub-Saharan African countries. Rather than large variations across countries, we found that 81% of the variation in competence is within countries and the characteristics of health care providers do not explain most of this variation. Professional qualifications—including cadre and education—are only weakly associated with competence: across our sample, one-third of nurses are more competent than the average doctor in the same country and one-quarter of doctors are less competent than the average nurse. Finally, while younger cohorts do tend to be more competent, perhaps reflecting improvements in medical education, it would take 25 decades of turnover to improve care by 10 percentage points, on average, if we were to rely on such improvements alone. These patterns necessitate a fundamentally different approach to health care human resource management, calling into question typical staffing policies based on qualifications and seniority rather than directly measured quality.

## Introduction

The most pressing problem in global health today is quality of care, not access: 91% of the world's population lives within a 1-hour drive of a health facility,^1^ but even patients with straightforward conditions are frequently misdiagnosed and incorrectly managed.^2-4^ While the research to date establishes the extent of severe quality deficits in multiple countries,^5^ what is currently missing is a deeper understanding of the variation in quality and whether low average quality is still consistent with the availability of some very high-quality providers in every context. This gap is notable, as evidence from even a relatively high-performing country such as the United States shows that there is tremendous practice-quality variation within seemingly homogeneous groups.^6^ Here, as a first step towards addressing that gap, we establish the extent of variation in the medical competence of health care providers using comparable measures across 11 sub-Saharan African countries.

Our data source is The World Bank's Service Delivery Indicators (SDI), which was started in 2010 to provide information on multiple aspects of human resource readiness in health systems. The SDI is based on representative in-person surveys of health facilities sampled from a national registry, with data on a range of indicators including the availability of medicines and facility staffing and absenteeism.^7^ Crucially for our purposes, the SDI initiative also completed clinical vignettes with health care providers. As we show, these clinical vignettes provide valid and reliable measures of the medical competence of health care providers for the tracer conditions that were assessed.^8-11^ In administering these surveys, the SDI included all types of health care workers—not just “doctors”—leading to comparable competence measures for providers across a wide range of professional cadres and educational qualifications.

Relative to alternate methods of collecting data on quality of care, clinical vignettes offer several advantages.^2^ First, in low-income contexts, patient charts are frequently inadequate or missing, severely limiting the utility of chart abstraction. Second, unlike data based on real patients, medical vignettes are standardized across providers and thus offer a measure of quality that is not confounded by differences in patient- or case-mix. Third, because the same vignette can be administered to health care providers regardless of their role, they allow for comparable measures across providers with different titles and training. Fourth, because the underlying health conditions are known to the researcher, actions can be benchmarked against existing national and international standards of care. Multiple studies show that measures of competence based on vignettes are a reliable measure of provider competence, and that they capture the best possible care that a patient could expect to receive when visiting a health care provider.^9-11^

The SDI microdata we use allow us to assess the medical competence of 16 127 primary health care providers from 8872 facilities using nationally representative samples in 11 sub-Saharan African countries, making this the largest study to date on medical competence. We produced a comprehensive analysis of provider skill in each country and the association between professional cadres, age, and competence. Relative to previous use of these data,^7^ our focus on variation illustrates that averages can be highly misleading in this context; in general, we document substantial variation in medical competence within countries, qualifications, and seniority, with significant implications for health policy.

### Context

The 11 countries in our sample are highly varied along multiple dimensions. For instance, Niger has a GNI (gross national income) per capita of 1510 US dollars (USD) in purchasing-power parity terms compared to USD 5680 in Kenya.^12^ In the most educated country, Kenya, 57% of children are enrolled in secondary schools compared to 24% in Niger.^13^ Infant mortality in these countries ranges from a low of 28 per 1000 in Kenya to a high of 78 per 1000 in Sierra Leone,^14^ and total health spending as a fraction of GDP (Gross Domestic Product) ranges from 3.4% in Nigeria to 8.8% in Sierra Leone.^15^

These countries also differ in how they structure their medical education and health delivery systems, an issue we discuss in detail in Section 1 of the Supplemental Appendix. For instance, in Kenya and Tanzania, professional cadres are similar to those in the United Kingdom, from where the professional titles used in these countries are originally derived: A “doctor” is a general practitioner with at least an undergraduate degree (typically a Medical Officer), “nurses” hold undergraduate nursing degrees, and a range of paraprofessionals support these cadres.^16,17^

However, responsibilities of someone in the “doctor” cadre—as well as their requisite educational qualifications—are not consistent across the other countries in the sample. For example, Madagascar and Niger consider many equivalent courses to be postgraduate, such that a “doctor” must hold an undergraduate degree in addition to a specific qualification.^18,19^ In Nigeria, by contrast, due to the severe shortage of highly formally educated staff, a titular “doctor” is typically responsible for the oversight and management of multiple facilities in a local government area, while the actual heads of facilities are typically nurses with a Master’s-type professional degree, and frontline staff are paraprofessionals.^20,21^ The actual equivalence of responsibility might place a Malagasy M.D. on the same level as a Kenyan undergraduate doctor and as a Nigerian M.S. nurse.

The composition of each workforce is also highly variable in terms of origin and qualification. Nigeria and Malawi report that as much as 40% of their recorded medical school graduates have emigrated as part of a medical “brain drain.”^22,23^ Niger does not have a medical school at all and care is provided by a combination of doctors working with NGOs (nongovernmental organizations) such as Médecins Sans Frontières and faith-based organizations, the few migrants the country attracts, and a few who could travel abroad for medical education and then returned.^19^ In Malawi, a large contingent of providers trained in South Africa are practicing, due partly to a combination of proximity, a lack of better employment opportunities, and visa restrictions on traveling elsewhere.^24^ In Nigeria, the local workforce has ratcheted up responsibilities as described above, such that Nigerian “nurses” appear to have similar responsibilities as “doctors” in, say, Kenya.^21^ Finally, there is no information in many settings on the work of non–degree-holding staff who may not be formally allowed to practice medicine, but are fortunately included in the SDI surveys and are especially common in Guinea-Bissau (where they are often “nurses”), Malawi, Nigeria, and Sierra Leone.

The diverse experiences and structures of these countries have thus produced wide variation in the provenance and content of the medical education required to officially hold a given cadre title as well as how those titles are mapped to official responsibilities. The key advantage of the survey-based direct measures of medical competence we use is precisely that they allow for comparable measures across countries, allowing us to better understand how the capacity of these medical systems might be evaluated with respect to quality.

## Data and methods

The data used for this study came from the SDI health surveys conducted by the SDI program at The World Bank in 11 sub-Saharan African countries between 2013 and 2019. For each SDI survey, the national Ministry of Health provided a complete listing of health facilities that offered primary care services. The sample of facilities for each country was generated by the SDI program team through random sampling from that listing of health facilities, after stratification by geography (rural or urban) and level (hospital, clinic, or health post). Section 2 of the accompanying Supplemental Appendix provides complete details of the sampling and survey process.

The SDI surveys collected information on individual health care providers through two steps in the facility survey process. In the first step, data on the facility were collected through a survey administered to the facility manager during site visits. As part of this survey, information was also collected on clinical staff, including their age, gender, educational qualifications, and professional cadre. The SDI program then categorized health care providers into (1) those who possessed at most a “Certificate,” which meant some kind of basic occupational training but no formal medical education; (2) holders of a “Degree” obtained through formal medical education such as nursing or undergraduate general medical education; and (3) “Advanced” education, typically a specialization degree. The SDI program also categorized each provider according to their professional cadre as a “Doctor” (including clinical officers), “Nurse,” or “Other (Para-Professional)” according to the facility roster. “Other” providers with a degree or advanced qualifications were re-coded as doctors; these were primarily medical professionals with advanced degrees such as radiologists and pharmacists. Supplemental Appendix Table 1 reports each professional classification that was reported in each country during the roster recording and indicates how those were mapped to the cadres used here.

In the second step, up to 10 health care providers who reported routinely seeing outpatients at each facility were randomly sampled for competence evaluation using clinical vignettes. The requirement that providers routinely saw outpatients was due to the emphasis on primary care in the SDI. Sampling therefore excluded providers who only participated in support services, inpatient management, or surgical care. Comparing staff rosters with the vignette sample showed that the SDI surveys included 97% of doctors, 70% of nurses, and 55% of other staff. The limit on the total number of health care providers at each facility only excluded providers in the 5% of facilities where over 10 providers reported seeing outpatients. In 95% of facilities, all such providers were included. Table 1 reports the breakdown of all health care workers who completed the medical vignettes by facility characteristics (rural/urban, public/private, and whether they practiced in a hospital, clinic or health post) as well by professional cadre, educational qualifications, gender, and mean age.

**Table 1.** Provider summary statistics for vignette sample.

	(1) Guinea-Bissau	(2) Kenya	(3) Madagascar	(4) Malawi	(5) Mozambique	(6) Niger
Facility characteristics						
Rural	59.2%	73.7%	44.6%	62.5%	81.0%	46.8%
Public	100.0%	62.2%	65.9%	55.0%	100.0%	88.6%
Facility type						
Hospital	4.9%	13.1%	11.3%	27.9%	39.7%	25.4%
Clinic	95.1%	23.0%	73.3%	67.1%	6.6%	37.0%
Health post	0.0%	63.9%	15.3%	5.0%	53.7%	37.5%
Provider type						
Doctor	17.5%	40.1%	56.7%	35.0%	50.1%	22.6%
Nurse	73.8%	58.5%	41.4%	24.9%	41.4%	57.7%
Other	8.7%	1.4%	1.9%	40.2%	8.5%	19.7%
Provider education						
Advanced	1.9%	8.4%	58.0%	1.6%	8.4%	61.5%
Diploma	21.4%	76.8%	38.9%	52.2%	87.4%	17.1%
Certificate	76.7%	14.8%	3.1%	46.2%	4.1%	21.5%
Provider gender						
Men	51.5%	50.3%	47.0%	66.9%	47.1%	40.4%
Women	48.5%	49.7%	53.0%	33.1%	52.9%	59.6%
Provider mean age, y	38.0	36.9	43.5	36.9	32.7	35.0
Number of observations	103	4485	619	1519	683	594

	(7) Nigeria	(8) Sierra Leone	(9) Tanzania	(10) Togo	(11) Uganda	(12) Total
Facility characteristics						
Rural	72.4%	58.6%	57.6%	63.4%	81.4%	63.5%
Public	93.6%	88.7%	69.3%	81.6%	66.8%	79.2%
Facility type						
Hospital	21.9%	10.1%	14.4%	15.7%	2.2%	17.0%
Clinic	64.4%	26.2%	25.8%	34.7%	43.2%	45.1%
Health post	13.7%	63.7%	59.7%	49.5%	54.6%	37.9%
Provider type						
Doctor	15.4%	19.1%	73.2%	22.8%	25.6%	34.4%
Nurse	16.7%	31.8%	16.4%	47.6%	50.5%	41.9%
Other	67.9%	49.1%	10.4%	29.6%	23.8%	23.8%
Provider education						
Advanced	26.9%	4.1%	6.8%	28.3%		20.8%
Diploma	5.6%	16.5%	59.7%	31.2%		39.9%
Certificate	67.5%	79.4%	33.5%	40.5%		39.3%
Provider gender						
Men	37.2%	24.0%	62.0%	44.6%	39.1%	46.4%
Women	62.8%	76.0%	38.0%	55.4%	60.9%	53.6%
Provider mean age, y	40.6	41.1	42.5	38.1	34.5	38.2
Number of observations	5017	829	1018	527	733	16 127

This table reports the descriptive statistics of all the providers that participated in the medical vignettes. *n* = 16 127 health providers across the 11 countries.

The competence of the sampled health care providers was assessed for up to 7 different tracer conditions.^9,10^ The vignettes used were as follows: childhood diarrhea with dehydration, childhood pneumonia, adult tuberculosis, adult diabetes mellitus, childhood malaria with anemia, neonatal asphyxia, and postpartum hemorrhage. These conditions were chosen because they constitute a substantial share of the estimated disease burden in sub-Saharan Africa.^25^ In each vignette, the provider was presented with an opening statement by the enumerator detailing the patient's complaint. The provider was able to ask any history questions and conduct physical exams, for which the enumerator would describe any relevant findings that would result. Then, when the provider indicated that they completed the history questions and examinations, the enumerator would ask for a diagnosis; finally, the enumerator would ask the provider what the correct treatment for the case would be. The information from provider rosters was combined with these vignette results to construct a combined dataset of 16 127 health providers practicing at 8872 health facilities.

Using the clinical vignettes, we constructed our first primary measure of quality, a provider-level “competence score” based on the completion of an essential condition-specific checklist of history questions and examinations across all vignettes, weighted using the psychometric methods of Item Response Theory (IRT).^9,11^ The resulting competence score was a continuous variable that was standardized to have an expected mean of zero and standard deviation (SD) equal to 1. The standardization implies that, across the full sample, the top fifth-percentile provider had a competence score of +1.5 SD and the top 10th-percentile provider had a competence score of +1.1 SD. Statistical details and validation are detailed in Section 6 of the Supplemental Appendix.

The competence score allowed us to examine the variation in competence within country and cadre in our results. We calculated means, medians, and percentiles, including the interquartile range (IQR), for the distribution of competence scores in various provider subgroups of interest, which were used to produce the distributional analyses presented in the figures and results. We also calculated the average competence score for each professional cadre in each country, and at the same competence score in other subpopulations, the corresponding provider's percentile rank.

The second two primary measures of quality assessed the health care provider's performance in diagnosing and managing the clinical vignettes. The diagnosis and management performance of each provider were binary variables that took the value of 0 when incorrect and 1 when correct. These were coded at the vignette level for 112 889 individual vignette responses, with up to 7 vignettes per provider. To assess whether the case was correctly managed, the SDI program applied internationally harmonized best practices across all settings using the information recorded in the vignettes.^25^

We then used ordinary least squares regression analysis to quantify predictors of those three primary outcomes: (1) the competence score of each provider estimated from all vignettes, (2) whether providers diagnosed each of the vignette conditions correctly, and (3) whether providers indicated the correct management for each of the vignette conditions. We successively added predictors to our models, estimating 3 regression models for each outcome. First, we regressed each outcome on individual demographics (professional cadre, age, and education) only. Second, we regressed each outcome on individual demographics as well as on facility characteristics (facility level and whether the facility was rural or urban). Third, we regressed each outcome on individual and facility characteristics, and included a binary control variable for each country to adjust for cross-country differences (these coefficients are not reported). We also calculated the proportion of variation explained by each of these combinations of covariates as the *R^2^* of the regression model.

## Results

### Variation in competence and performance among providers

**Figure 1.**
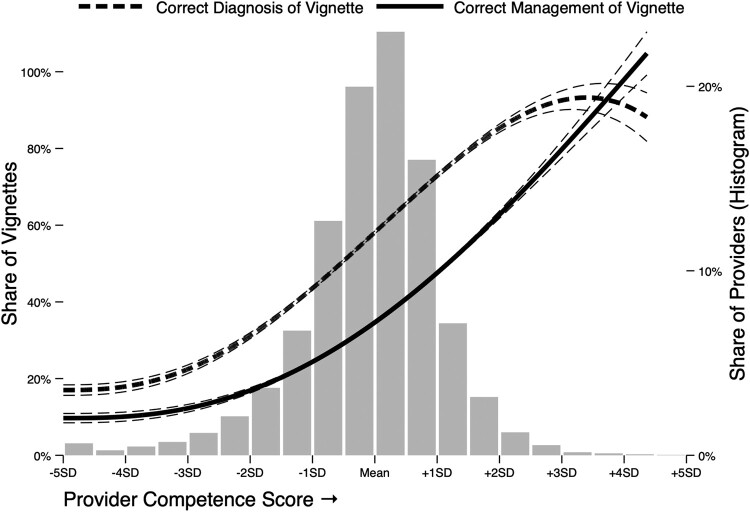
Provider Item Response Theory (IRT) competence scores and vignette performance. This figure illustrates the expected performance in vignettes in terms of correct diagnosis and correct management (left vertical axis) as a function of the provider's IRT competence scores (horizontal axis). The dashed line is a polynomial fit of the share of providers with the indicated competence score correctly diagnosing a randomly selected vignette. The solid line is a polynomial fit of the share of providers with the indicated competence score correctly managing a randomly selected vignette. The light dashed lines are the 95% CIs for the fitted relationships. All observations are weighted, such that the sum of weights for each country is equal. Observations are top-censored in the fitted lines at a competence score of +4.5. The right vertical axis indicates the share of providers whose competence scores fell into each bin indicated by the histogram, with bin width of 0.5 SD.

Figure 1 illustrates the distribution of the competence score, as a histogram, as well as the associations between the competence score and the likelihood of correct diagnosis and management in the vignettes. Since the questions about diagnosis and management were asked after the IRT items were completed, and both were excluded from the construction of the competence score, these associations provide a valid test of the predictive power of the competence score for subsequent diagnosis and management. Supplemental Appendix Figure 1 and Table 3 provide results showing the correct management of vignettes in every subpopulation of providers in every country, as predicted by the competence scores.

The mean provider in the sample correctly diagnosed 60% of vignettes and correctly managed 35%, but the substantial variation in competence implied that providers with competence scores below −1 SD (15% of the sample) could treat no more than 1 in 5 vignettes correctly, while those with competence scores above +1.5 SD (5% of the sample) could treat 3 in 5 vignettes correctly. The competence score was strongly associated with the provider's ability to correctly diagnose and manage the vignette conditions, with an increase of 1 SD in a provider's competence score associated with, on average, a 10-percentage-point (p.p.) increase in the frequency of both correct diagnosis and of correct management in vignettes.

**Figure 2.**
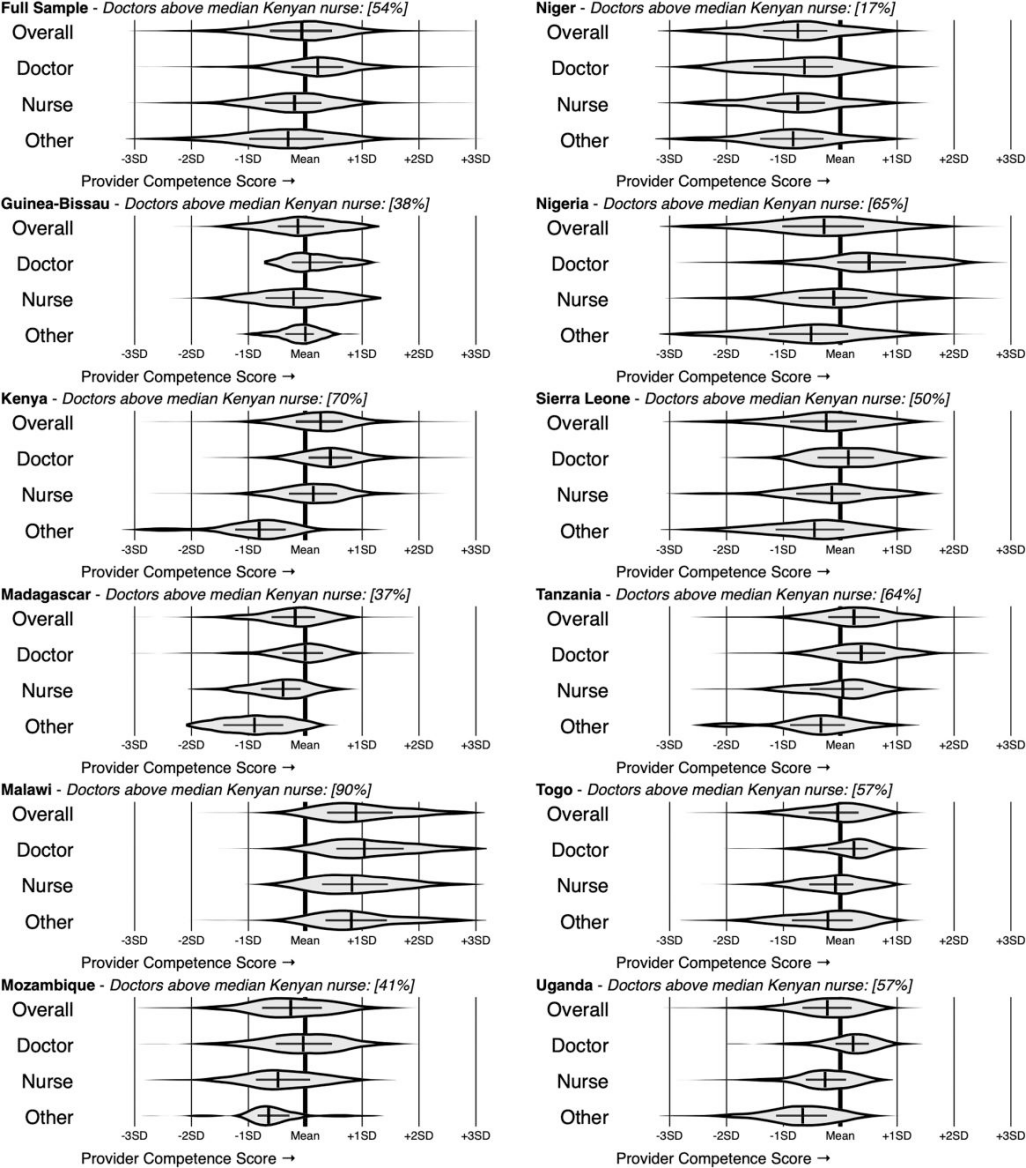
Provider competence scores by country and professional cadre. Each violin plot depicts the distribution of the Item Response Theory (IRT) competence scores for each professional cadre in the full sample for each country (censored at ±3). The vertical lines indicate the median of each competence score distribution; the horizontal lines indicate the IQR; and the density distribution is represented by the height of the shaded region. Each title indicates the share of doctors in the country with a higher competence score than the median nurse in the Kenya sample. The “Doctor” cadre includes any non-nurse with a completed degree or advanced education. The “Other” paraprofessional cadre is defined as any other non-nurse. In the full sample, all observations are weighted, such that the sum of weights for each country is equal.

Figure 2 decomposes the overall distribution of provider competence scores across countries and professional cadres, illustrating the median, the IQR, and the full distribution. Countries could be classified into three broad groupings differentiated by the competence of the median provider in the country relative to the median provider in the full sample. Malawi, Kenya, and Tanzania constituted the “high-performing” group, where the median provider had a competence score of +0.5 SD and could correctly manage 40% of vignette cases. Togo, Madagascar, and Guinea-Bissau constituted the “middle-performing” group, where the median provider had a competence score of −0.2 SD and could correctly manage 35% of vignette cases. Niger, Nigeria, Sierra Leone, and Uganda constituted the “low-performing” group, where the median provider had a competence score of −0.5 SD and could correctly manage 30% of vignette cases.

These cross-country differences, however, were observed to be small relative to the variation in competence within each country. In Nigeria, for example, the median competence was the second-lowest overall across the 11 countries. However, the top 25% of providers in Nigeria outperformed the top 25% of providers in every country outside the “high-performing” group; and the top 1% of providers in Nigeria outperformed the top 1% of providers in all countries except for Malawi. Every country had providers in both the top and bottom 10% of the entire sample; the average within-country IQR spanned the same competence range as the mean difference between the high- and low-performing country groups.

We investigated whether this massive variation in competence within countries arose solely from differences in the competence of health care providers with different professional titles. If this were the case, we would have expected to find large differences in competence scores for health care providers of different cadres, and similar scores for health care providers in the same cadre. Indeed, in all countries, doctors had the highest median competence scores, and in all countries (except for Guinea-Bissau), other paraprofessionals had the lowest.

However, just as when examining the within-country variance, there were high- and low-performing health care providers in every cadre in every country. In fact, the best and the worst scores within every cadre in every country were typically among the top and bottom 10% in the overall sample. Consequently, instead of wide differences across cadres, there was significant overlap in competence scores for providers of different cadres. For example, a significant proportion of nurses were more competent than the average doctor in each country, ranging from 23% in Uganda to 50% in Niger, with an average of 31% of nurses being more competent than the average doctor.

Combining the variation in competence within country with the aggregate differences across countries shows that the competence of a doctor can vary widely across countries. We used the median performance of a nurse in Kenya, with a competence score of +0.14, as an arbitrary reference, reporting the share of doctors in each country who outperformed this subset. In the full sample, the competence of 54% of doctors was higher than the median Kenyan nurse and across countries; this ranged from 17% in Niger to 90% in Malawi. Supplemental Appendix Figure 1 and Table 3 provide additional results showing that this wide variation occurs in every subpopulation of providers in every country.

### Predictive power of demographics for competence and performance

To quantify these associations and provide confidence intervals, Table 2 reports regression estimates of the demographic correlates of medical competence. As expected, doctors and nurses outperformed paraprofessionals on the competence score measure in the full sample (models 1, 4, and 7), by 0.67 SD (95% CI: 0.57–0.77 SD) and 0.17 SD (95% CI: 0.08–0.26 SD), respectively. Doctors and nurses were correspondingly 17 (95% CI: 15–19) and 7 (95% CI: 5–9) p.p. more likely to diagnose vignettes correctly and 11 (95% CI: 8–13) and 4 (95% CI: 2–6) p.p. more likely to manage vignettes correctly.

**Table 2.** Regression results for competence score and vignette performance.

	Provider competence score			Diagnosed vignette correctly			Managed vignette correctly		
	(1)	(2)	(3)	(4)	(5)	(6)	(7)	(8)	(9)
Provider type (vs other)									
Doctor	0.67	0.52	0.28	0.17	0.14	0.07	0.11	0.08	0.07
[95% CI]	[0.57, 0.77]	[0.41, 0.62]	[0.18, 0.39]	[0.15, 0.19]	[0.12, 0.17]	[0.04, 0.10]	[0.08, 0.13]	[0.06, 0.10]	[0.04, 0.09]
Nurse	0.17	0.10	0.07	0.07	0.06	0.05	0.04	0.03	0.05
[95% CI]	[0.08, 0.26]	[0.01, 0.19]	[−0.02, 0.16]	[0.05, 0.09]	[0.04, 0.08]	[0.02, 0.07]	[0.02, 0.06]	[0.01, 0.05]	[0.03, 0.07]
Provider age									
Per 10 years	−0.03	−0.04	−0.04	0.00	0.00	0.00	0.00	0.00	0.00
[95% CI]	[−0.05, −0.01]	[−0.06, −0.02]	[−0.06, −0.02]	[−0.00, 0.01]	[−0.01, 0.01]	[−0.01, 0.01]	[−0.00, 0.01]	[−0.01, 0.00]	[−0.01, 0.00]
Education (vs certificate)									
Advanced degree	−0.26	−0.37	0.33	−0.04	−0.06	0.10	−0.04	−0.05	0.05
[95% CI]	[−0.35, −0.16]	[−0.47, −0.27]	[0.23, 0.43]	[−0.06, −0.02]	[−0.08, −0.04]	[0.07, 0.13]	[−0.06, −0.02]	[−0.07, −0.03]	[0.02, 0.07]
Diploma	0.02	0.01	0.09	0.04	0.03	0.05	0.02	0.01	0.02
[95% CI]	[−0.07, 0.11]	[−0.08, 0.10]	[0.01, 0.18]	[0.02, 0.06]	[0.01, 0.05]	[0.02, 0.07]	[0.00, 0.04]	[−0.01, 0.03]	[0.00, 0.04]
Facility level (vs health post)									
Hospital		0.53	0.29		0.07	0.07		0.05	0.05
[95% CI]		[0.46, 0.60]	[0.22, 0.35]		[0.05, 0.09]	[0.06, 0.09]		[0.04, 0.07]	[0.04, 0.06]
Clinic		0.35	0.14		0.00	0.03		0.02	0.02
[95% CI]		[0.30, 0.40]	[0.08, 0.19]		[−0.01, 0.02]	[0.02, 0.05]		[0.01,0.03]	[0.01, 0.04]
Facility characteristics									
Urban		−0.18	−0.05		−0.01	0.00		−0.02	−0.02
[95% CI]		[−0.23, −0.12]	[−0.11, 0.01]		[−0.03, 0.00]	[−0.02, 0.01]		[−0.03, −0.00]	[−0.03, −0.00]
Private		0.45	0.07		0.07	−0.01		0.08	−0.01
[95% CI]		[0.40, 0.51]	[0.02, 0.12]		[0.05, 0.08]	[−0.02, −0.00]		[0.07, 0.09]	[−0.02, 0.00]
Country indicator controls	No	No	Yes	No	No	Yes	No	No	Yes
Number of observations	14 951	13 604	13 604	96 591	87 344	87 344	92 034	84 015	84 015
Regression *R^2^*	0.065	0.117	0.304	0.023	0.024	0.071	0.009	0.012	0.056

This table reports results from linear regression models with the competence score, management accuracy, and diagnostic accuracy as dependent variables (the first is at the provider level; the others are at the level of the vignette). Each dependent variable was included in a regression specification estimating 3 different models. First, a provider-characteristics model is reported that includes only the provider's professional cadre, educational level, and age. Next, a facility-characteristics model was estimated including the facility's care level, whether it was urban and whether it was private. Finally, a regression was estimated including these variables and also including country indicator variables. The model details for each specification are reported in the lower panel of the table. All observations are weighted, such that the sum of weights for each country is equal. Regression sample sizes in columns 1–3 are subsets of the total sample of 16 127 providers where covariates are missing for individual observations; Uganda is excluded from all specifications since all data on education are missing there. Sample sizes in columns 4–9 are reported at the vignette level of observation, with up to 7 vignettes per provider.

However, the relative advantage of doctors and nurses was attenuated as we successively included facility characteristics (models 2, 5, and 8), as providers in hospitals and private facilities had higher competence scores, as did those in rural settings. When we included country indicator controls (models 3, 6, and 9), the competence gap between doctors and others shrunk even further. Since the regression coefficient was biased upward without those controls, and because being a country with more doctors implied a higher likelihood that an individual is a doctor, we inferred that there was also a positive relationship between average competence scores and the share of doctors at the country level. Conversely, advanced degrees were negatively correlated with competence in the full sample, but when we included country indicator controls the coefficient was positive as expected within each country, suggesting that countries with high shares of providers with advanced degrees had lower average competence scores.

We also assessed whether medical competence was higher in recent (younger) cohorts of medical professionals by including age as an additional independent variable. Across all outcomes in all models, we estimated that younger cohorts had higher competence than older cohorts, with a difference of +0.04 SD per 10 years of age (95% CI: 0.02–0.06 SD). Since a 1-SD increase in competence was associated with a 10-p.p. increase in correct management, we concluded that, under current patterns, it would require 250 years of new provider cohorts to increase average correct management by 10 p.p. above current levels, corresponding to the non–statistically significant and near-zero effects reported in models 4–9.

**Figure 3.**
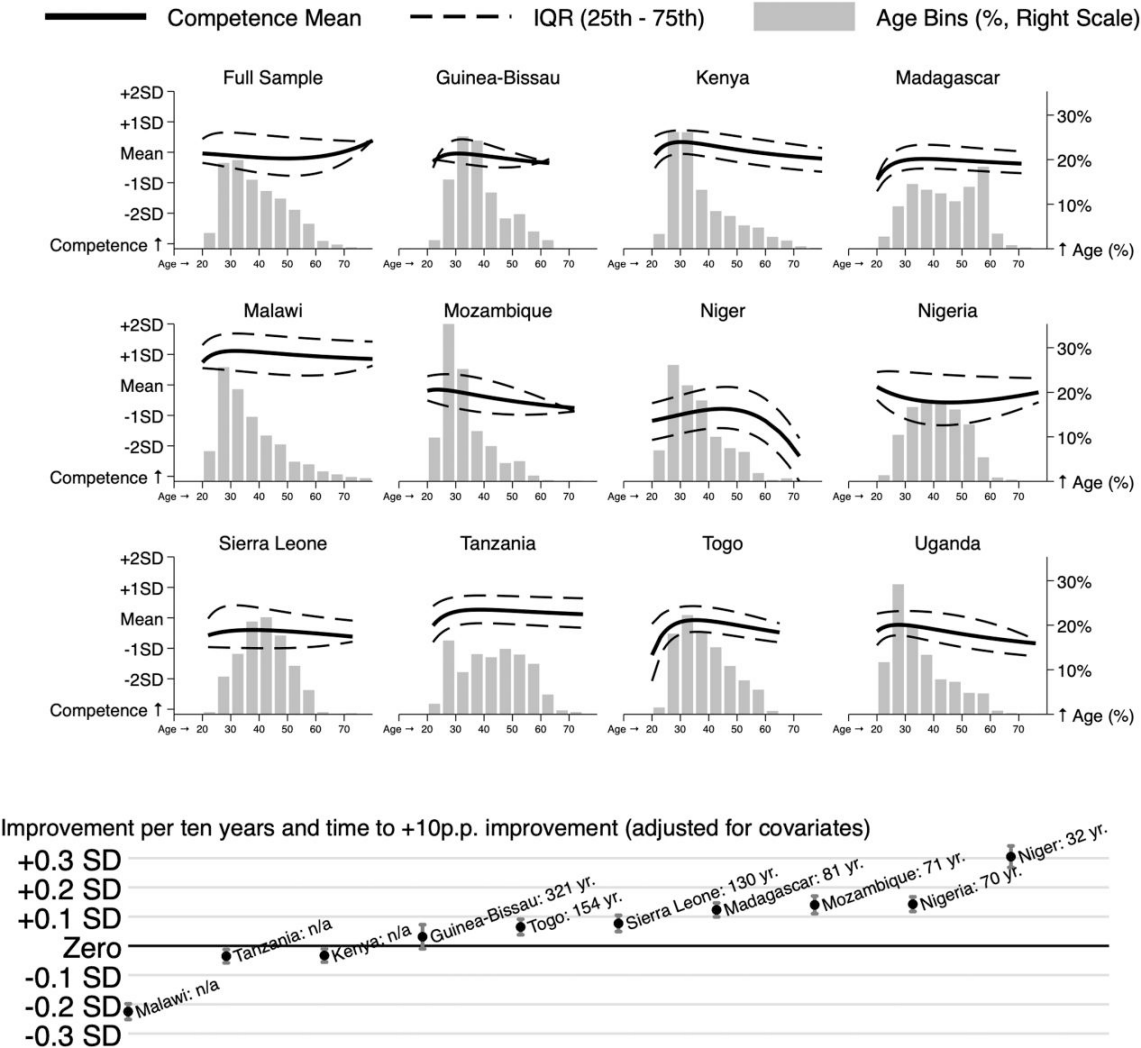
Provider competence scores by age and country. Each panel shows the distribution of provider age in the full sample or the specified country (histogram with 5-year age bins; right axis). The overlaid solid lines report a fractional polynomial estimated fit of the mean competence score for providers at each age. The dashed lines report fractional polynomial estimated fits of the 25th and 75th percentile of competence scores at each age. In the full sample, all observations are weighted, such that the sum of weights for each country is equal. Abbreviation: n/a, not applicable; p.p., percentage point; yr, years.

In order to examine the correlation with age in greater detail, Figure 3 reports the relationship between medical competence and age separately for each country, with the age distribution provided as a histogram. In countries that had seen a large expansion of training capacity in recent years, we would expect to see larger cohorts of younger doctors, as we do in Mozambique, Malawi, Kenya, and Uganda (although migration in very high numbers could generate similar patterns). Then, we report the number of years required to increase average competence by 1 SD for each country, or “n/a” for the cases where younger cohorts perform worse.

The time it would take to increase the average competence score by 1 SD under business-as-usual improvements ranged widely, from 321 years in Guinea-Bissau to 32 years in Niger. Since competence scores were lower in newer cohorts in Malawi, Tanzania, and Kenya, in these countries business-as-usual improvements would not lead to any gains over time. We were interested to observe that there was no clear association between the relative size of recent cohorts and their competence scores relative to older cohorts. In both Malawi and Niger, for example, younger cohorts were substantially larger, suggesting an increase in training capacity. But, while in Malawi the competence of comparable recent cohorts had declined relative to older groups by about −0.2 SD per 10 years, in Niger it had improved by +0.3 SD. Kenya, Togo, and Guinea-Bissau also evidenced larger young cohorts, but showed little change over time in their competence scores. Thus, some countries appeared to have expanded the availability of training without any relative decline in competence, while others had maintained the same competence without any significant expansion in the size of recent cohorts. Others, like Tanzania, neither expanded the size of young cohorts nor achieved higher competence scores among younger providers.

Even though the correlation between quality measures and provider characteristics is of interest, one of the most striking results is that none of these regression models could explain much of the variation in the competence score as measured by *R^2^* statistics. The provider-characteristics model alone explained just 6.5% of the variation in competence scores and the model with provider and facility characteristics explained 11.7%. The inclusion of country indicator controls in the model increased the explained variance to 30.4%, which implies that the country in which the health care worker is practicing in explained almost 3 times as much of the competence of health care workers (18.7%) as individual characteristics did. These results confirmed that, for all combinations of cadre, educational attainment, age, and facility characteristics within each country, 70% of the variation in provider competence was not explained and 81% of variation occurred within countries.

## Discussion

It is now widely accepted that universal health coverage must provide “access with quality.” Nevertheless, systematic data on quality from large, multicountry samples remain sparse. This study combined unique data on clinical vignettes administered to 16 127 health care providers across 11 sub-Saharan African countries through The World Bank's SDI program with IRT to produce detailed analyses of provider-level competence scores within and across countries, professional cadres, and age cohorts.

Our analysis demonstrated the value of data such as these, which allowed us to study practice-quality variation at a granular level. Consistent with previous studies that have uncovered severe deficits in knowledge, we also found that 15% of providers in our sample could correctly manage only 1 in 5 vignettes. However, rather than universal deficits in competence, the most striking feature of these data is the tremendous variation in quality. Every country in the SDI sample had providers with high and low competence scores and, in most subpopulations, the bottom 15% of providers could correctly manage, at most, 1 in 5 vignettes, while the top 10% of providers could correctly manage over half. The wide variation in competence that we uncover was not due to differences in provider or facility characteristics, which jointly explained only 11.7% of the variation in competence. Instead, within every cadre and facility type, there were high- and low-performing providers, implying that it is very difficult to predict the performance of an individual using demographic or facility characteristics with reliable accuracy.

One particularly notable result was the weak and variable association of competence with age. The correlation between age and competence captures two potentially countervailing forces. On the one hand, if health care providers become more competent with experience, older cohorts should be more competent than younger cohorts. On the other hand, if the quality of training improves, younger cohorts will be more competent than older cohorts. Distinguishing the relative importance of the two channels requires data on the same cohort of health care providers at two different points in time, which is currently not available.

Nevertheless, the literature allows us to make an educated guess as to which of these forces is stronger. In particular, systematic reviews indicate that performance declines as providers age: “empirical studies evaluating the relationship between clinical experience and performance suggests that physicians who have been in practice for more years and older physicians possess less factual knowledge, are less likely to adhere to appropriate standards of care, and may also have poorer patient outcomes.”^26^ If experience effects are negative, as the systematic review suggests, then the fact that young cohorts performed only marginally better than older cohorts in most settings must imply that the average quality of medical education for new cohorts has likely declined over time: Older cohorts likely completed their education with higher competence scores than younger cohorts did. These results have several policy implications that we discuss next, but prior to doing so, we highlight three methodological limitations in our analysis.

### Methodological limitations

The first limitation is that this overview of medical competence across the 11 countries in the SDI does not focus on the complex and particular challenges of individual countries; nor does it do complete justice to the variety of educational qualifications, cadres, and different types of providers across countries in our sample. There are significant differences in the challenges and opportunities of medical education in Nigeria, where questions of emigration and medical admissions are paramount,^27,28^ versus Niger where there are no medical colleges and doctors are trained abroad, versus Malawi where the College of Medicine that trains 70% of medical graduates was opened only in 1991.^29^ We hope that the techniques and methods developed here spur interest in individual country studies and we are committed to providing both the data and the code used for this paper publicly to aid such efforts.

The second limitation is that the SDI data allow us only to focus on medical competence as a measure of quality. While these are indeed the right measures of what health care providers know, and are closely related to the quality of medical training, multiple studies show that health care providers are less likely to correctly diagnose and manage a patient in their clinical practice than in tests of medical competence.^4,30^ Further, the SDI surveys measure competence for 7 tracer conditions, and it could be that measures are sensitive to the conditions that are chosen. Both of these limitations call for more exploration and measurement of medical competence and clinical practice moving forward; such measurements need to become part of the regular functions of regulatory institutions rather than one-off ad hoc efforts.

The third limitation is that, on the technical front, constructing appropriate provider-level weights for statistical aggregation of these type of data remains challenging. These data were collected using a sampling strategy that allows for facility-level weighting; measures using this weighting therefore reflect averages across randomly selected facilities. However, such sampling weights do not reflect (1) the share of patients who seek care at any given facility or (2) the relative workload and staff time or responsibilities of any given provider within a facility. More rigorous weighting schemes may be required moving forward. In order to precisely clarify the roles of various providers in any setting and the distribution of patient loads across provider cadres and specific providers, detailed time-motion studies at individual facilities offer a promising avenue for future research. The Supplemental Appendix provides illustrative information on the roles of various professional cadres in each of the study countries.

### Policy implications

With these limitations in mind, we now turn to the policy implications. We focus on the consequences of the variation in competence for (1) the measurement of quality, (2) the design of compensation systems, and (3) quality-improvement strategies.

### Quality measurement

These results call into question the use of health care provider demographics, including professional cadre, educational qualification, and seniority, as standard indicators of the availability of high-quality care at population levels. Frequently used measures of human resource availability, such as “the number of doctors per 1000 population,” are essentially uninformative in sub-Saharan Africa when it comes to assessing a population's access to high-quality care because they do not accurately predict the medical competence of those health care providers. A clear implication of our results is that such indicators confound, rather than clarify, the situation regarding the availability of high-quality care and should be used with considerable caution and appropriate caveats.

### Compensation systems

In addition, for providers who are public employees, which is the large majority in these countries, compensation and promotion are often based on educational qualifications and seniority. As these two measures were very weakly correlated with medical competence, it must mean that compensation and competence are also weakly linked in these health systems. The weak association between qualifications, age, and competence has been pointed out previously,^9^ but this is the first time that a sufficiently large and representative sample from a range of sub-Saharan countries and providers has shown that this issue is pervasive in every setting and subpopulation.

The granular individual-level data presented here, if collected regularly as part of performance appraisal systems, could potentially lead to higher-quality care. For one, governments could improve the allocation of providers across facilities and geographies. For instance, they could ensure that the most competent providers are assigned to facilities with higher patient loads, with potentially higher compensation accompanying greater workloads. Individual assessments could also provide incentives for health care providers to invest in core medical competencies, knowing they will be rewarded in the future. Current systems of remuneration unfortunately reward characteristics that are only weakly correlated with the ability of providers to correctly manage patients.

### Quality-improvement strategies

The weak and variable associations with age also make clear the challenge that countries face as they try to simultaneously augment capacity and improve competence. There are two parts to this challenge. In countries that are not rapidly increasing capacity, the health care providers who have already completed their training will form the bulk of the workforce for several years or decades to come. Therefore, aggregate improvements in competence can only come from continuing medical education of health care providers who have already graduated. Unfortunately, how to improve competence among existing providers remains a vexing challenge, as the common approach of updating medical competence through short courses and targeted interventions has not led to the at-scale systematic improvements that are needed.^31^

In other countries like Malawi, there has been a large expansion in the capacity of the health system and here, investments in upgrading the training infrastructure may lead to rapid dividends—for instance, through the systematic deployment of Ministry of Health's standard treatment guidelines for care.^32^ A first step towards improving the quality of training is to assess competence among recent graduates and provide information on the quality of different training institutions. Conducting similar vignette studies at degree-granting institutions with new graduates will allow these methods to be scaled-up in a cost-effective manner, since the single greatest expense in these SDI program studies was the travel and time cost of visiting health facilities across large geographies.

In conclusion, the combination of IRT competence scores with the detailed data collected through the SDI program at The World Bank provides a unique and informative look at medical competence among health care providers in sub-Saharan Africa. Instead of a uniform message of severe quality deficits, the results point to substantial unexplained variation within countries, cadres, and cohorts. They revealed that policymakers cannot infer the availability of high-quality care for populations from provider demographics. There is an urgent need for individual, context-specific examination of how education, experience, and health system factors shape individual provider competence, and to reduce the reliance on “cadre and qualifications” as measures of the availability of quality medical care. These results lead to important new questions about the ability of health systems to identify, allocate, and reward existing high-quality providers, as well as to measure and improve the quality of medical education for future provider cohorts.

## Supplementary Material

qxae066_Supplementary_Data
