# Supplementary material for: The medical competence of health care providers in sub-Saharan Africa: Evidence from 16 127 providers across 11 countries
Source: Health Aff Sch. 2024 Jun 7;2(6):qxae066. doi: 10.1093/haschl/qxae066 (PMC11157171; doi:10.1093/haschl/qxae066)
Supplement: qxae066_Supplementary_Data [file qxae066_supplementary_data.zip › appendix.pdf]

Supplemental Appendix

The Medical Competence of Healthcare Providers in Sub-Saharan Africa: Evidence from in-person vignette tests of 16,127 providers across 11 countries

Benjamin Daniels[1], Jishnu Das[1,2,*], Roberta Gatti[3], and Andres Yi Chang[3]

[1]Georgetown University, Washington DC, USA
[2]Center for Policy Research, New Delhi, India
[3]The World Bank
[*]Correspondence to: jishnu.das@georgetown.edu

ABSTRACT

Despite a consensus that quality of care is critically deficient in low-income countries, few nationally-representative studies provide quality measures that are (a) comparable across countries and (b) provide estimates of quality variation within and across countries. To address this gap, we used nationally-representative data from in-person clinical vignettes measuring the competence of 16,127 healthcare providers in 11 sub-Saharan African countries. Rather than large variations across countries, we found that 81% of the variation in competence was within countries. The characteristics of healthcare providers did not explain most of this variation. Professional qualifications – including cadre and education – were only weakly associated with competence. As a result, even though doctors were more competent than nurses in general, a significant proportion of nurses were more competent than the average doctor in the same country, ranging from 23% in Uganda to 50% in Niger. Finally, while younger cohorts tended to be more competent, it would take 250 years of turnover to improve correct management by 10 percentage points. These patterns necessitate a fundamentally different approach to healthcare human resource management, calling into question typical staffing policies based on qualifications and seniority in favor of directly measured competence and quality.

1 Health system contextual descriptions

Guinea-Bissau. Ranked 175th by the Human Development Index (HDI), the 2 million residents of this primarily Guinea-Bissau Creole speaking country (and former Portuguese colony) are served by a total of 125 Health Centers (equivalent to health clinics) in the country, and eight hospitals. One quarter of the population lives in the capital, Bissau. All facilities are publicly-run, and all except one hospital were surveyed by the SDI team. Specialized providers are extremely rare and degree-qualified providers are uncommon; for example, Médecins Sans Frontières (MSF), which ended many programs there in 2020, reports that the country's medical training program does not offer pediatric training and the number of pediatricians in the country numbers in the single digits.[1] Annual health spending per capita is estimated at USD$165, or about 8.4% of GDP.[2,3]

Kenya. Ranked 143rd by HDI, with a population of 55 million, Kenya has a diverse system of public, private, and not-for-profit health facilities. The National Bureau of Statistics reported 9,654 such facilities classified into 6 service levels. This survey excluded Level 1 (communities) and Levels 5 and 6 (secondary and tertiary referral hospitals); leaving dispensaries (the predominant form of facility, listed here as health posts), health centers (clinics), and county referral hospitals. There are large inequalities among the country's 47 provinces and between its rural and urban regions; just 6% of physicians practice in rural areas.[4] Private facilities and higher-level facilities are overwhelmingly located in denser areas, while sparsely-populated locales rely on public and not-for profit services at lower care levels. Kenya has a well-educated workforce where most health workers – including nurses – are typically required to have completed a full degree program in order to practice formal medicine. Specialists are less common and are concentrated in laboratories and hospitals; para-professionals are very rare.[5] Annual health spending per capita is estimated at USD$203, or about 4.3% of GDP.[2,3]

Madagascar. Ranked 164th by HDI, Madagascar's two medical schools require a seven-year doctoral curriculum in order to practice medicine as a doctor,[6] leading to a very high proportion of its health workers reported as having advanced education; nearly all nurses have diplomas and there are nearly no para-professional health workers reported. In 2016, there are 3,491 facilities serving an island population of around 28.4 millions. About one-third of facilities are private and they are mainly concentrated in urban areas. "CSB1" facilities have no doctor (health posts); "CSB2" facilities have a doctor (clinics); and central district hospitals handle more advanced cases. Annual health spending per capita is estimated at USD$59, or about 3.9% of GDP.[2,3]

Malawi. Ranked 174th by HDI, the 19.7 million residents of Malawi are served by 1,106 health care facilities, split roughly equally between public and private (including not-for-profit, NGO, and faith-based services). Malawi has one medical school which had trained a total of 254 doctors by 2007; however, it is estimated that nearly 40% of graduates have left the country.[7] A large share of the country's working doctors have been trained abroad in places such as South Africa.[8] To fill the remaining service delivery gap, para-professionals with less than degree-education make up a plurality (41%) of primary health care providers. Clinics and health centers make up the majority of the health

system, and despite staff shortfalls, Malawi has the second-largest share of “hospitals” across all countries sampled due to the official categorization of a large number of rural referral locations as hospitals. Annual health spending per capita is estimated at USD$82, or about 5.4% of GDP.[2,3]

Mozambique. Ranked 181st by HDI, Mozambique’s 32.2 million citizens are served by some 1,600 medical facilities in a publicly-run health system and an unknown number in private practice. Mozambique has four structural levels of health care providing three intensities of health care.[9] The data collected only includes two primary levels of health care: first level hospitals and health centers (included here as health posts; a few intermediate facilities were qualified as clinics but these were very rare compared to other countries). There is one private medical school and two public medical schools; specialized nursing education is a relatively recent development.[10] About one-third of trained doctors are believed to emigrate.[11] Annual health spending per capita is estimated at USD$99, or about 7.6% of GDP.[2,3]

Niger. Niger remains the very lowest-ranked nation (189th) by HDI, with 25.1 million residents. Most facilities are publicly owned and organized into three levels of care, with “private” facilities primarily being not-for-profit or faith-based. There is no tertiary education in general medicine available in the country, although the legislature has recently advanced proposals attempting to establish one.[12] Doctors are consequently very scarce, and nursing staff account for most of the medical workforce (often with post-graduate education in nursing, reflected in the high share of “advanced” degrees among the workforce). Annual health spending per capita is estimated at USD$79, or about 6.2% of GDP.[2,3]

Nigeria. Ranked 161st by HDI, is the most populous country in Africa with more than 211.4 million residents, but features high degrees of inequality and heterogeneity across its regions and its dozens of major ethno-linguistic groups (more than 500 languages are spoken in the country) as well as between religious groups such as Muslim and Christian populations. Health facility management is de-centralized but largely public, with the federal government managing tertiary care, the 36 state governments managing secondary “general hospitals”, and the 774 local government areas (LGAs) managing primary care (assessed in this survey). There are extensive concerns about emigration of trained doctors to other English-speaking countries; the country’s 42 medical schools produce around 3,000 MD-equivalent graduates annually against a national pool of around 40,000 graduates.[13] This dynamic leads to extensive reliance on non-degree auxiliary staff, especially at the locally managed primary care level; within each LGA each medical doctor supervises a group of facilities staffed by nurses and community health workers.[14] Annual health spending per capita is estimated at USD$173, or about 3.4% of GDP.[2,3]

Sierra Leone. Ranked 182nd by HDI, the country’s 8.1 million residents are served by a primarily public system. The basic package of essential health services (BPEHS) in the country is delivered through both primary and secondary levels of healthcare. The country has two medical schools; they produce very few MD equivalents, and MSF estimates that around 7% of health staff were lost in the 2014-2016 Ebola epidemic.[15] As a result, nurses and especially midwives make up the bulk of medical staff; certified nurses and

doctors typically hold post-graduate education while others do not. Staffing is relatively lower-skilled in rural areas; according to SDI reporting, no rural area had access to higher level facilities than health posts, and none had formal doctors present. Annual health spending per capita is estimated at USD$147, or about 8.8% of GDP.[2,3]

Tanzania. Ranked 163rd by HDI, the 61.5 million residents of Tanzania are served by a health system that is approximately two-thirds publicly provided and one third private (including not-for-profit). Annual health spending per capita is estimated at USD$40, or about 4% of GDP.[2,3] It is structurally similar but significantly poorer and more rural than neighboring Kenya; while advanced-degree-holding doctors make up a relatively small share of providers nationally according to local definitions, the cadre is substantially over-represented in the SDI sample due to (a) oversampling in the capital (Dar es Salaam) and other urban areas and (b) the classification of the many diploma-qualified clinical officers and their assistants as "doctors" in this sample.

Togo. Ranked 167th by HDI, Togo's 8.5 million residents. Togo has dispensaries, health centers, and district hospitals, all of which were included in the SDI survey; about 80% are publicly-run. Doctors are required to have both a masters' and a doctoral degree, making a total of 7 years of education necessary for formal practice; specialization is beyond this.[16] As a result, doctors and clinical officers comprise about 10% of providers, and nurses and midwives make up the rest of the health workforce. Annual health spending per capita is estimated at USD$102, or about 3.8% of GDP.[2,3]

Uganda. Ranked 159th by HDI, Uganda has 47.1 million people. There are five medical colleges and approximately 3,000 health facilities, of which 70% are publicly-run. Unfortunately, the SDI survey of Uganda did not collect information on educational standing; although 45% of medical students reported attempting to emigrate in one survey.[17] Annual health spending per capita is estimated at USD$95, or about 4.0% of GDP.[2,3]

2 SDI sampling approach

The SDI surveys were designed to obtain indicators of the availability of health personnel that are accurate and representative of primary health facilities at the national level. When possible, further levels of representativeness were targeted to allow for representative disaggregated analysis (such as state-level) in addition to valid national-level statistics. The sampling strategy for SDI surveys, therefore, generally followed a multistage sampling approach. The main units of analysis were primary health facilities and providers (e.g. doctors, nurses, facility managers, etc.). Such a multistage sampling approach was used to divide the selection of sampling units – health care facilities – from large potential populations of sampling units in a step-by-step fashion. For example, SDI sample strategies were, dependent on context, designed to allow for meaningful representative analysis across sub-regions, rural and urban areas, public and private providers, and other relevant features such as facility level. Stratification, therefore, improved the precision of indicators and allowed for more accurate within-country comparisons.

To achieve this result, the sampling frame (the list of all eligible health facilities with

basic information on facility characteristics) was typically obtained from the national Ministry of Health or the relevant National Statistics Office. Given the surveys' focus on primary care, facilities at all levels of care – typically categorized as hospitals, health clinics, and health posts (or the national equivalent) – were included *if* they were reported to provide outpatient primary care services. As a result, specialized hospitals and clinics or those that only provided complex inpatient care were generally excluded from the sampling frame. From this initial listing, a final sample frame for each context was generated by using relevant additional national data sources (such as registries of private clinics, where appropriate) and validating the list entries to avoid missing entries, duplicates, and outdated or incorrect information. At that point, the sample frame was also categorized into strata as necessary for the representative sampling appropriate to the national context.

Once the final frame was complete for each country, a first stage selection of sampling units was carried out independently within each stratum. Often, the primary sampling units (PSU) for this stage were geographical cluster locations (e.g. districts, communities, counties, neighborhoods, etc.). These were randomly drawn within each stratum with a probability proportional to the size (PPS) of the cluster (typically measured by the location's number of facilities or providers). Once locations were selected, a second stage took place by randomly selecting facilities within the selected locations (either with equal probability or with PPS) as secondary sampling units. When needed, replacement facilities were drawn in each location for health facilities that no longer existed, were not functional, refused to participate, or were inaccessible due to security concerns. These replacement facilities were selected in keeping with the probability sampling approach. Clear replacement protocols were also established ex-ante to avoid bias in the results, and replacement facilities were not allowed to be chosen on the basis of logistical ease.

Sampling procedures were also adapted to country-specific contexts. In a few instances, certain types of facilities were excluded from the sampling frame. For instance, in the 2013 Health SDI in Nigeria, tertiary facilities were excluded from the surveys as there were very few of them (11 across 6 states) and they would not be appropriately surveyed with the same instrument used for primary or secondary facilities. Likewise, in Guinea-Bissau (2018) and Mozambique (2014), the objectives of the exercises combined with the absence of a comprehensive sampling frame, logistical, and budgetary constraints, resulted in the exclusion of the private sector from the sample. Finally, for the 2014 Health SDI in Tanzania, 93 facilities (approximately 1.2% of the total universe of health facilities) were deleted from the frame because they corresponded to facilities that did not provide primary health services such as larger regional hospitals, dental clinics, specialized clinics, or facilities which served prison populations.

Likewise, some countries needed to modify the sampling process due to logistical and budgetary restrictions. For instance, in the 2014 Health SDI surveys in Mozambique, the sample was reduced from originally 300 facilities to 204 health facilities due to logistical and financial problems. However, these exclusions were carefully managed by a sampling expert and did not affect the representativeness of results at the national level. Similarly, in Nigeria 2013, the health SDI was designed to survey only 12 states out of 36, mostly

due to the size of the country and budgetary restrictions. The surveys are, nonetheless, representative for each of the selected states.

Finally, some locations or facilities were favored for selection as they were deemed unique in the sample or their characteristics represented opportunities for interesting comparisons or analysis. For instance, this was the case for the 2018 Health SDI surveys in Sierra Leone, where all hospitals and health centers were selected within stratum, while other facility types like clinics and health posts were randomly sampled. Overall, SDI sampling procedures were planned and protocoled ahead of data collection activities. Contextual decisions to non-randomly select or eliminate certain geographic locations or facility types are taken ex-ante and in manners that do not compromise rigor and representativeness of results, as well as comparability within objective stratum and across countries.

This study used equal weights for all providers within each country equal to 1 divided by the number of providers surveyed in that country; in aggregate analyses, we weighted all countries equally. Using the data unweighted was inappropriate because of the large differences in sample size: Nigeria and Kenya together make up nearly two-thirds of the observations, while the Tanzanian sample is one-quarter the size of the Kenyan sample. It would be possible to conduct population-weighted estimates for pooled statistics using either the number of patients seen by each provider or the country populations. However, the Nigerian data would dominate any estimates under such an approach (with about 210 million people, followed by Tanzania with some 60 million and Kenya with some 54 million). Finally, SDI provides weights, which are not standardized across countries, but using them does not substantially affect any of our estimates.

3 Country-specific facility sampling

The information in this section is reproduced almost verbatim from the respective SDI reports. Additional information is available in the original SDI reports for each country. Publicly available reports can be found in the SDI website (https://www.sdindicators.org/countries). Unfortunately, not all SDI reports are currently publicly available. We recognize that these reports and the excerpts reproduced here contain, in some instances, some minor mathematical errors. Furthermore, small differences are evident between the initial values and sample sizes contained in the reports and the corresponding data shared by the SDI team after additional cleaning and aggregation were completed.

3.1 Guinea-Bissau

The SDI survey in Guinea-Bissau visited a total of 132 of the 133 public health facilities reporting a total staff of 2338 of which 1522 are health workers. The number of health facilities per region varies between 8 facilities in Biombo and 21 in the region of Cacheu. Over two thirds (62%) of health facilities in the country are located in rural areas and a majority are categorized as Type C health centers which are in turn distributed across the different regions. Guinea-Bissau has a total of 7 hospitals of which 5 are Regional Hospitals located in the Region's main city while the two reference hospitals are located in the city of Bissau and in Biombo.

3.2 Kenya

Administratively, Kenya's health system is divided into 47 counties. In each county, the health sector is and managed by a County Health Management Team (CHMT). The Sampling Frame used is a list of health facilities provided by the Ministry of Health. The list contains a total of 9,654 facilities, with geographic identifications of County, constituency, sub-county and ward; as well as ownership status such as Public or Private. In addition to the list, facility type such as dispensaries, clinics, health centers, and hospitals; with their location in either Rural or Urban was provided by the nation's statistical agency - Kenya National Bureau of Statistics (KNBS). It is important to note here that the sampling strategy for the SDI in Kenya was done by the KNBS. The list of facilities to include was sent to the National Council For Population and Development (the agency responsible for the data collection) by KNBS.

The SDI survey collected information from 3,094 health facilities and 13,026 health providers located in 47 counties. The results are representative at the following levels:

- National, urban and rural levels
- Indicators were representative at the county-level as well
- Government and private (both non-for-profit [*Original Footnote: This includes faith-based and NGOs facilities.*] and for-profit) facilities
- Representative of services at the first, second and tertiary facility levels, which includes: Dispensaries/clinics, health centers, and hospitals (including first- and tertiary-levels hospitals).

The procedure was as follows:

- The sample frame included 9,631 facilities (both public/private and at 3 levels of care) in 47 counties.
- In each of the 47 counties, a target number of facilities was decided based on the overall/combined number of facilities available in the county as per the sampling frame [*Original Footnote: Excluding tertiary hospitals.*]
- Once this target number of facilities per county was decided, it was distributed proportionally to the number of facilities across each of the strata generated by the permutation of ownership type (i.e. community/public/private non-profit/private for-profit) AND level of care (i.e. dispensary/clinic, health center, and first level hospital).

3.3 Madagascar

The sampling for Madagascar 2016 SDI has been undertaken by the Malagasy national statistical office (INSTAT). The sampling frame used for the Madagascar health SDI was the 2014 list of health facilities obtained from the services of the Ministry of Public Health (MinSANP) before the start of the field work. The original sample frame contained 3,491 health facilities with geographic identifier variables such as region, district, commune and

even GPS coordinates. The sample frame was then purged of duplicates and facilities for which there were missing values. This process left INSTAT with a final sample frame of 3,415 health facilities.

Although the SDI is usually representative at the national and urban and rural areas, in Madagascar it was requested that the survey be also representative of the following strata: (1) Provinces, (2) private/public, and (3) urban/rural areas.

The stratification variables provide the domains (strata) and reporting levels of the survey. The stratification also depends on the most important indicators to be measured in the survey (input availability, absence rates, and diagnostic accuracy). Finally, it is advisable to order the clusters within each stratum by variables that are correlated with key survey variables for further implicit stratification when systematic selection is used. The ownership of the facility is one such key variable.

A multi-stage clustered sampling strategy is adopted. The first stage cluster selection is carried out independently within each stratum. The primary cluster considered is the district which is therefore the primary sampling unit (PSU). For each province, the capital district is drawn exhaustively, and four other non-capital districts have been drawn sans remise with unequal probability, taking into account the size of the districts in terms of number of primary schools and health facilities. As a result, a total of 30 districts have been sampled. Health facilities were randomly drawn with equal probability as a secondary sampling unit. At the third stage, health workers are selected.

3.4 Malawi

The Malawi Harmonised Health Facility Assessment, a combination of a Service Availability and Readiness Assessment (SARA) and the Service Delivery Indicators (SDI) surveys, was a census of all health facilities in Malawi. The survey assessed 1,106 facilities in Malawi between November 2018 and March 2019. A total of 12,773 health care workers were recorded and a sample observed for absenteeism, 1,433 health workers were assessed with clinical vignettes, and 4,100 clients were interviewed (2,333 mothers regarding health care for their children under-five years and 1,785 pregnant women receiving ANC).

The Malawi master facility list identified a total of 1,224 health facilities. Of the 1,224 facilities, data collection was completed for 1,106 facilities. Data collection was not completed for 118 facilities which were either not located or no longer existed.

Out of the 1,106 facilities surveyed, 101 were hospitals, 492 were health centres, and 513 were dispensaries, clinics, and health posts. Of these, 575 were public facilities and 531 private facilities, including 165 Christian Health Association (CHAM) and 53 NGOs. The majority of the health facilities were located in rural areas (760). In terms of regional distribution, 208 facilities were located in the North, 399 in the Centre, and the remaining 499 in the South.

3.5 Mozambique

The survey used a multi-stage, cluster sampling strategy which allowed for disaggregation by geographic location (rural and urban), and facility type (health centers; and first level

hospitals). A total of 204 randomly selected health facilities comprising 166 health centers and 38 hospitals were included. In the process 658 and 1,116 health professionals were assessed for competence and effort, respectively. The results are representative of Mozambique as a whole as well as by level of facility and location (rural/urban). The sample was reduced from originally 300 facilities due to logistical and financial problems, and provide a representative snapshot of the health services environment in public facilities in Mozambique.

The sampling strategy was a simple random sample using the stratification detailed above. However, during fieldwork, the sample was reduced from originally 300 facilities to 204 health facilities due to logistical and financial problems. The sample was originally drawn for those 300 facilities and the necessary sample reduction was done after a third of the sample had already been surveyed. To decide which additional facilities were to be sampled, we used the following decision criteria: For each open stratum, we decided to close the stratum if the resulting standard error was lower than the resulting standard error from randomly sampling using the above mentioned methodology.

Replacement facilities were drawn from each location in case the sampling frame included health facilities that no longer existed, were not functional, or were inaccessible due to security concerns. Note, these back-up facilities were not to be used for logistical ease. Replacement health facilities were selected in keeping with the probability sampling approach.

3.6 Niger

The Niger SDI survey collected information from 256 lower-level health facilities and 1,355 health providers. The survey covered facilities around the entire country, with the exception of a few areas. The region of Diffa was in a state of emergency declared by the Government of Niger during the survey and was excluded as were the areas of Tesker, Tilia, Tassara (localized risks), and Bilma (few facilities, very great distances).

The sample frame was constituted from administrative data (health management information system; two sources) and survey data (Service Availability and Readiness Assessment sample frame) on facilities provided by the Ministry of Health and census data provided by the National Statistical Institute (Institut National de la Statistique; INS). The team de-duplicated facilities in the administrative data and worked to properly identify their locations within administrative boundaries to define the sample frame.

The sample frame is the set of health posts, health centers, and hospitals that could be geographically identified, that met certain comparability and inclusion criteria set with the Ministry of Health, and that were in areas that were feasible. For reasons of comparability the ministry excluded all private facilities that offered surgery and all hospitals that were not normal district hospitals. The feasibility constraints excluded the region of Diffa, where the government had declared a state of emergency, and Tassara, Tesker, and Tillia (security) and Bilma (geographic isolation).

The sample frame was stratified along rural/urban (per the INS definitions), ownership (public/private), and facility type (Health post/Health center/District hospital) to maximize intragroup homogeneity. The sample was designed based upon the request of the ministry

to include health posts, which represent the overwhelming majority of health facilities, and the need to have sufficient sample sizes for health centers and hospitals. Selection was done with probability proportional to the population served by the facilities.

In the Niger health system, multiple types of facilities exist; of these the Health posts (case de santé), clinics (centres de santé intégré) and district hospitals (hôpital de district) were included in the survey population. These facilities account for the nearly 87 percent of the health service utilization as reported in the 2014 household survey undertaken by the National Statistical Office (Institut National de la Statistique).

The surveyed population comprised three types: Health posts (poste de santé), health centers (centre de santé intégré), and district hospitals (hôpital de district). The survey used a two-stage sampling strategy that allowed for disaggregation by geographic location (rural and urban), by provider type (public and private), and by facility type. Since there were 28 first-level district hospitals, a decision was made to sample 16 of them, which represents a 57 percent sampling fraction. There were 8 facilities where second visits were not undertaken in accordance with the SDI methodology. These facilities, and their staff, are excluded from the absence rate calculations.

3.7 Nigeria

The health facility survey covered 2,480 rural and urban health facilities across twelve states, including 2,298 public providers and 182 private facilities. The survey also included 12,678 health providers measured for absence. The results provide a representative picture at the state level of the quality of service delivery, and the physical environment within which services are delivered at the three levels of health facilities: health posts, health centers, and the first level of hospitals.

The target population was the population of 12 selected states in Nigeria (Anambra, Bauchi, Bayelsa, Cross River, Ekiti, Imo, Kaduna, Kebbi, Kogi, Niger, Osun and Taraba). Four data sources were used in developing the sampling frame: (i) Public facilities: Ministries of Health; (ii) Location-specific data on the fraction of the local population living in poverty was obtained from the Nigeria national statistical authority; and (iii) The fraction living in urban areas, was obtained from the national statistical authority. This note assumes that the sampling frame provided by the Ministry of Health is complete, and that the poverty data are the latest available. Population estimates were obtained from the latest population projections provided by the National Population Commission (NPC), using the latest census data. There are numerous types of facilities. The facility list was restricted to three major categories: Health Posts; Health centers (including medical clinics); First-level hospitals. Taking ownership into account, the facilities were then aggregated into six categories.

Based on the most recent available data from national statistical authority at the time, the facilities were categorized as rural or urban and poor on non-poor. These two binary distinctions yield four strata within which to sample facilities. Within each stratum, facilities are selected randomly.

Anambra, Bauchi, Ekiti, Niger, Cross-Rivers, Kebbi. Sampling for each of these states was done separately. Within each state, facilities were stratified by type and by

urbanization. Urbanization fell in the same three categories as described above for all other states; facility type was primary, secondary, or tertiary. Three exclusion criteria were applied. There were very few tertiary facilities (11 across these 6 states), and they would not be appropriately surveyed with the same instrument as would be used for primary or secondary facilities. As such, the focus was only on primary and secondary facilities. The second exclusion criterion was that 60 facilities were dropped, 10 in each state, because they had been used in pre-sample piloting. Finally, private health facilities were not included. In this restricted sample frame, there were very few secondary facilities in each state, so a census of these facilities was conducted. The aim was to visit a total of 200 facilities in each state, and after doing a census of the secondary facilities (between 14 and 27 in each state), the remainder of the sample was divided proportionally across up to three urbanization strata in each state, according to the number of facilities in each stratum. The result was that the probability of being sampled varies across states, but within a state, does not vary a great deal for primary public facilities. All secondary public facilities were sampled with probability one.

Bayelsa, Imo, Kaduna, Kogi, Osun, Taraba. The plan here was to sample 1,260 facilities across the six states, so an average of 210 per state. This involved a series of decisions. First, with only 208 public facilities in Bayelsa, it was decided to do a census of public facilities in Bayelsa. Second, with only 174 secondary facilities outside Bayelsa, it was decided to do a census of secondary facilities outside Bayelsa as well. Third, there were 2,133 private facilities across the six states. A decision was made to sample 150 of these. There is a primary/secondary level distinction in the data for these facilities, but the meaning is not very clear. As such, a state-level strata for the private facilities was constructed, and the team sampled approximately proportionally to stratum population. These three decisions accounted for 208+174+150=532 facilities, leaving 728 for public primary facilities in five states. Weighing the options (varying total public facility counts with fixed public primary facilities per state, versus varying public primary facility counts with fixed total public facility counts), it was decided that the precision of state-public numbers was more important than the precision of state-public-primary numbers, so the total number of public facilities per state was set at 180, and the team sampled however many public primary facilities were needed to arrive at that number given that all secondary facilities had been included. In keeping with previous health facility sampling in Nigeria, the team stratified the sample on a ternary urbanization variable, and sample approximately proportionally to population (oversampling one small cell in Imo state for numerical reasons).

3.8 Sierra Leone

The SDI survey interviewed 547 facilities across Sierra Leone, 1,700 workers were observed for absenteeism and 818 health workers were assessed with clinical cases. There were 32 hospitals, 99 health centers and 416 health posts. Within the sample, there were 501 public facilities and 46 private facilities across Sierra Leone. Further, there were 382 rural and 165 urban facilities. The data collected are also representative of the districts, urban and rural areas strata.

Administratively, Sierra Leone's health system is divided into 14 health districts. Each health district is divided into chiefdoms, and managed by a District Health Management Team. The Sampling Frame used is a list of health facilities provided by the Ministry of Health and Sanitation. The list contains a total of 1,300 facilities, with geographic identifications of Region, District and Chiefdom; as well as ownership status such as Public or Private. In addition to the list, facility type such as health posts, health centers, clinics, and hospitals; with their location in either rural or urban as provided by the nation's statistical agency.

The study was intended to be conducted in all 14 health districts; but however, in two of the 14 districts, i.e. Kailahun (87 facilities) and Koinadugu (78 facilities) a census was done; while in the remaining 12, a survey was conducted. Therefore, the sampling frame excludes the list of facilities for the two census districts, which gives a total of 1,135 facilities.

The sample for this SDI is a stratified sample selected in two stages from the sampling frame. Stratification is achieved by separating the list of facilities into ownership (public or private), urban and rural areas. In total, 36 sampling strata have been constructed, since there are no private facilities in the rural areas. Samples will be selected independently in each stratum, by a two-stage selection. In the first stage, facilities will be selected randomly. An implicit stratification and proportional allocation will be achieved at each of the lower administrative levels by sorting the list, within each sampling stratum, according to lower administrative units. Also, in order to have a gain in precision, facilities such as hospitals and health centers will be selected and assigned a probability of 1 within each sampling stratum.

The sample allocation took the precision consideration at domain level into account. The allocation was done in two steps: firstly, a proportional allocation was used to allocate the target number of facilities to each study domain; then the domain sample size was proportionally allocated to each sampling stratum (that is, the ownership, urban and rural areas) within the domain.

The sampling for Sierra Leone SDI was undertaken by Statistics Sierra Leone (SSL): the Sierra Leonean national statistical office. The sampling frame used for the Sierra Leone health SDI was the list of health facilities obtained from the MoHS before the start of the field work. The original sample frame contained 1,300 health facilities with geographic identifier variables such as region, district, and chiefdom.

3.9 Tanzania

The sampling frame the Tanzania health SDI used, was the 2012 list of health facilities obtained from the services of the Ministry of Health and Social Welfare (MoHSW) before the start of the fieldwork. The original sample frame contained 7,472 health facilities with geographic identifier variables such as region, the division, the ward and even the street. This sample frame was merged with the list of wards from the most recent 2012 census to obtain the size of the population a specific facility is serving which will be later used as a weight for selecting facilities. The sample frame was then purged of 899 facilities, which were not functional because they were either closed, or under construction. A

further 91 facilities were deleted from the frame because they were not eligible for the SDI i.e. regional hospitals, dental clinics, specialized clinics, etc. Two more facilities were suppressed because they served prison's population. This process left us with a final sample frame of 6,480 health facilities.

With 995 (15 percent) of health facilities with missing information on ownership (i.e. public/private), the sample frame had an important challenge to offer. Because there was no way to determine the ownership status of those health facilities before going to the field, the facilities were left in the frame but categorized as unknown for ownership. During the data collection the head of facility was asked whether their facility's ownership status and the data collected. This new information will be used for post-stratification adjustment.

Although the SDI is usually representative at the national and urban and rural areas, in Tanzania it was requested that the survey be also representative of the traditional strata in household surveys which are (1) Dar es Salaam, (2) other urban areas, and (3) rural areas.

The stratification variables provide the domains (strata) and reporting levels of the survey. The stratification also depends on the most important indicators to be measured in the survey (input availability, absence rates, and diagnostic accuracy). Finally, it is advisable to order the clusters within each stratum by variables that are correlated with key survey variables for further implicit stratification when systematic selection is used. The ownership of the facility is one such key variable.

A multi-stage clustered sampling strategy is adopted for the 2014 Tanzania SDI. The first stage cluster selection is carried out independently within each explicit stratum. The primary cluster considered is the district which is therefore the primary sampling unit (PSU). At the second stage health facilities will be selected and at the third stage health workers. It was decided than within each stratum, except Dar es Salaam, 25 districts would be chosen with probability proportional to size (population). Note that this implies that this stage each person in each stratum has an equal probability that her district will be selected.

3.10 Togo

The Togo SDI survey collected information from 180 lower-level health facilities and 1,364 health providers. In the Togolese health system, three categories/types of facilities can be identified, and these three types were included in the survey population. These facilities account for the overwhelming part of the health service utilization as reported in the 2011 household survey undertaken by the National Statistical Office (Direction générale de la statistique et de la comptabilité nationale).

The results provide an assessment of the quality of service delivery and the environment in which the services are delivered in rural and urban locations, in public and private (nonprofit) health facilities. While the private (nonprofit facilities) largely include facilities owned by faith-based organizations, there are also some facilities that are owned by nongovernmental organizations.

The surveyed population comprised three types: dispensaries (USP1), health centers

(USP2), and district hospitals (HD1 and HD2). The survey used a two-stage sampling strategy that allowed for disaggregation by geographic location (rural and urban), by provider type (public and private nonprofit), and by facility type. Since there were 20 first-level district hospitals, a decision was made to sample them exhaustively.

The sample frame was constituted from information provided by the Ministry of Health and the National Statistical Directorate (Direction générale de la Statistique et de la Comptabilité nationale; NSD). Problems with toponymy were addressed with the NSD and the Survey on Neonatal and Obstetrical Emergencies (SONU). The ministry provided two different facility lists, one that was supposed to be exhaustive from the census of all neonatal and obstetric care facilities (SONU; census of all facilities, then in-depth questions for those that did neonatal/obstetric care) and another from the Service Availability and Readiness Survey (100 facilities). Extensive efforts were deployed to address concerns related to toponymy (with the NSD) and to facility listings (with the ministry).

The sample frame was stratified along rural/urban (per the NSD's definitions), ownership (public/private), and facility type (USP1/USP2/HD1) to maximize intragroup homogeneity. An implicit stratification on the poverty rate of the area was done based upon the published poverty reports (2012). The selection was done with probability proportional to the population served using the Core Welfare Indicators Questionnaire's (2012) information on facility usage rates as the allocation criteria among facilities within a given area.

3.11 Uganda

The Uganda SDI survey was administered to 2,357 health providers across 387 health facilities. The survey used a multi-stage, cluster sampling strategy, which allowed for disaggregation by geographic location (rural and urban); by provider type (public, and private: for profit and non-profit) and by facility type (health posts, health centers and first-level hospitals).

The sampling frame for the 2013 Uganda SDI was based on the Health Facility Inventory of 2012 provided by the Ministry of Health. The original sample frame contained 4,999 health facilities (HC2, HC3, HC4, and hospitals) with identifier variables from the region to the parish in which each facility was located. The inventory also included the ownership of each facility as well as whether it was functional. The final sample frame was purged of all non-functional facilities as well as large hospitals which are not part of the SDI study. The final frame had 4,488 health facilities.

Although SDIs are usually representative of the national and urban and rural areas, in Uganda it was requested that the survey be also representative at the regional level. Because of its special status, Kampala was extracted from the Central region and considered a stratum in and of itself.

Unfortunately, the sample frame did not contain an urban/rural variable necessary for proper (implicit) stratification of the health facilities. With the help of the Uganda Bureau of Statistics (UBoS), further work was done to create an urban/rural variable and match each health facility according to its location. The stratification variables provided the domains (strata) and reporting levels of the survey. The stratification also depended on the most

important indicators to be measured in the survey (absence rates and performance). Finally, it was advisable to order the clusters within each stratum by variables that were correlated with key survey indicators for further implicit stratification when systematic selection was used. The main variable that was used was the facility ownership status (i.e. public/private).

Multi-stage clustered sampling strategy was adopted in the Uganda SDI. The first stage cluster selection was carried out independently within each explicit stratum. The primary cluster considered was at the county level, which was, therefore, the primary sampling unit (PSU). At the second stage, schools were selected and, at the third stage, health facility's staff for absence and clinicians for competence assessment. It was decided than within each stratum, except Kampala, 10 counties would be chosen with probability proportional to size (number of households).

4 SDI provider surveys

For provider interviews, a fixed number of health workers who have recently provided outpatient consultations were selected within each facility to provide information for the different questionnaire modules such as the clinical vignettes. In most facilities, all providers present during the survey were included; at very large facilities, a random sample of present providers was taken (this was required at fewer than 5% of facilities surveyed). Since SDI focuses on primary health care provision and health posts and clinics tend to be relatively small, most of the health workers in each facility are surveyed; only those not present were typically left out of the sample, meaning that each sample can be taken as representative of the daily availability of knowledgeable staff. In other words, we do not overestimate availability by assuming all staff are always available; and we therefore do not need to correct for absenteeism ex-post for this type of estimate.

SDI survey instruments are designed to collect information on three aspects of service delivery: (1) provider effort, (2) provider competence, and (3) facility inputs. Provider effort was measured by collecting health workers' absenteeism rates and caseload burden. Provider competence was measured using interactive "case scenarios" in vignettes to measure health providers' diagnostic and treatment accuracy, as well as adherence to clinical guidelines, and management of maternal and neonatal complications. Facility inputs were measured by observing each facility's equipment, medicine, and infrastructure availability.

SDI surveys required specialized coordination and specific enumerators. In particular, the patient case simulations ("clinical vignettes") required that enumerators had at least a basic understanding of medical knowledge. Health care practitioners that have recently provided care for patients responded to these clinical scenarios to assess their competence and skill in diagnosing and treating illnesses of high prevalence in low- and middle-income countries. At least two enumerators conducted the vignettes and received intensive training on a detailed set of instructions that must be followed during the interviews. Each enumerator was given a specific role to play: One enumerator acted as the patient and presents the health worker with the symptoms of each of the clinical

cases, while a second enumerator silently noted the questions and clinical procedures provided by the health worker.

Survey teams visited health facilities twice. First, during an announced visit, most of the information was collected, including a roster of all the medical providers. This visit was scheduled ahead of time and done with the head of the health facility, who also provided a roster of all the health workers in that facility. Then, during an unannounced visit, absenteeism rates were checked using 10 randomly selected health care workers from the main roster collected during the first visit.

When collecting the roster of medical workers, the medical education and cadre of each of them was also collected. Given that medical/health education and accreditation systems widely vary by country, the harmonization of these two variables into broader and comparable categories required manual attention to each listed category. Medical education was categorized into no formal training, holders of a "Certificate", holders of a "Degree", and holders of an "Advanced Degree". About half of the countries had categories that matched very closely one-to-one to this classification, and the remainder were categorized ex post. For instance, in Mozambique, nurses have "levels" and this characteristic was used to classify them into these categories: nurses of elementary level were classified into the "Certificate" category, nurses of basic and medium level went into the "Degree" category, and nurses of advanced level into the "Advanced" degree category. In Nigeria, 35 very specific categories were all mapped into these 3 categories, again with some level of judgment but following these general rules.

For the cadre classification, "Nurses" and "Doctors" were present in all locations and categorized as recorded. The reference category, "Para-Professional", was constructed by pooling all other providers in a single category, then removing those with "Degree" and "Advanced Degrees" from it (labeling them "Doctors"). That left only assistants, pharmacy aides, technicians, midwives and similar types of actually para-professionals of all types in this cadre category. **Table 1** shows these classifications in detail.

5 Vignette performance in sub-populations

Figure 1 presents a visual representation of the distribution of expected shares of correctly managed vignettes among healthcare providers across various sub-populations within each country. The figure highlights the performance levels of providers at the median, outer Tukey adjacent values, and the 25th and 75th percentiles (inter-quartile range or IQR) competence score distribution. **Table 3** provides numerical values of the means and IQR values for each sub-population within countries.

Notably, substantial decreases in the width of the competence score distribution as measured by the IQR were rare across the various sub-populations. High-performing and low-performing providers, relative to both the overall mean and the country-specific mean, could be found in nearly every demographic sub-population within each country. This finding suggests that provider competence is weakly associated with particular sub-populations, and that wide differences in performance levels exist within most sub-populations, regardless of the specific country or context.

6 Creation and validation of item response theory (IRT) competence scores

Item response theory competence scores were estimated using every provider's response rate to 241 possible history questions and examinations across each of the seven vignette conditions using the openIRT package in Stata 17 (Zajonc 2014; https://github.com/tristanz/OpenIRT). The IRT approach assumes that there is a single latent ability variable, "competence", that drives response patterns. To test that the data come from a unidimensional factor structure, we estimated component eigenvalues for the 57 vignettes history questions with at least 10,000 observations. The first panel in **Figure 2** shows that the eigenvalue for the first principal component (7.68) was 3.6 times greater than the second (2.12), consistent with the assumption of unidimensionality. The second panel illustrates how often each history question was asked, plotted against its contribution to the first principal components. There were no negative contributions or over-weighted elements, indicating that the completion of every checklist items indicates greater competence.

Using the information available from each provider's completion of these items where available (and appropriately accounting for missingness), each item is characterized based on discrimination, difficulty, and guessing parameters. "Difficulty" is defined as the competence score required for a provider to have had a 50% likelihood of completing the item; "discrimination" describes how quickly providers improve at an item as their competence increases. "Guessing" characterizes the intercept, or the likelihood that a provider with minimal competence (or, alternatively, a non-medical layperson) would complete the item. The IRT algorithm produced an estimated competence score for each provider based on the exact pattern of questions they asked and examinations they requested during the initial portion of the vignettes – that is, on their ability to ask questions and conduct examinations appropriate to the case before being given any further information about the true condition.

The 3PL IRT model is based on the Rasch model and assumes that the probability of a response r_{ij} on item j by examinee i is a function the examinee ability parameter θ_i and three item parameters: a guessing parameter c_j , an item difficulty parameter b_j (the ability score at which a provider is 50% likely to complete the item), and a discrimination parameter a_j (which characterizes how quickly providers learn to complete the item as their competence increases):

$$P(r_{ij}) = c_j + \frac{1-c_j}{1+e^{-a_j(\theta_i - b_j)}}$$

The 3PL IRT model allows for different levels of difficulty for each item, as well as different levels of discrimination for each item. This allows for the model to better capture the differences in difficulty and discrimination between different items. The parameters of the model are estimated using maximum likelihood estimation.

The estimated parametric fits are illustrated for the overall history question set and for demonstrative items in **Figure 3**, with a comparison between the theoretical IRT fit (line)

and the actual performance of providers (plotted points). We show both the predicted relationship between the competence score and the likelihood of asking each question (the solid line), as well as the observed data points averaged within 100 percentiles of competence.

Figure 4 illustrates the complete distribution of difficulty and discrimination for all items. For all checklist items, providers with higher levels of the competence score were more likely to complete the item in the vignette. The IRT method computes a level of "difficulty" for every checklist item, defined as the level of competence required to achieve a 50% probability of asking the item. There is a close correspondence between the difficulty computed using IRT and clinical concepts. For example, questions regarding the duration of diarrhea or pneumonia were "easy", and even providers with very low competence scores (below -2) asked them in 75% of cases. By contrast, providers with very low competence almost never asked a chest symptomatic about the nature of the cough, but for providers scoring above average (competence score > 0), the likelihood of asking increased to 75% or higher. Finally, asking a possible diabetic about lower limb numbness was a very "difficult" item that only providers among the top 5% asked 75% of the time or more.

As illustrated in **Figure 5**, low-competence providers completed very few of the items across all vignettes; high-competence providers completed a very high share of items. Total response rates were very consistent with the theoretical IRT curve, indicating a good fit of the data to the competence scores. Providers with moderate competence had more diverse response patterns. One key pattern is that in the range where most providers fell – between -2 standard deviations and +2 standard deviations – nearly all questions show substantial linear *improvement* as providers increase in competence. This is important because it implies that the value of improving the competence of the provider treating a given patient in the observed range of competence levels will correspond to well-defined improvements in care. By contrast, this is not true among very low-competence providers: between -4 and -2 standard deviations, for example, providers do so little that even a large gain in competence here is unlikely to reliably translate to quality improvements in practice.

Figure 6 and **Figure 7** show the estimated parametric curve for all items individually by vignette. **Table 4** lists the exact parameters estimated for each item. Items with very low required competence include very simple history questions such as "Asked the duration of cough" (for both TB and pneumonia) and exams such as taking temperature. High difficulty items included more advanced and invasive materials like "Checking for the Koplik sign" (malaria), "Completing a fundoscopy" (diabetes), or "Asking about past infections" such as measles or TB (for malaria, pneumonia, and TB). These patterns appear fundamentally sound and are therefore in line with intuitions about the level of medical competence required. Other high-difficulty items included physical exams that are often not asked by providers in hypothetical vignettes, such as measuring the height and weight of the patient or asking about diet, exercise, and risky behaviors – in general, those that are relatively unimportant for diagnosing these conditions.

By contrast, high-*discrimination* items included physical exams that often have direct

clinical relevance, such as taking pulse and respiratory rates. They also included things like asking HIV status of mothers and children and asking more detailed questions about the condition history. These included symptoms during pregnancy for PPH, asking about food and liquid intake for diarrhea, and family histories of diabetes. Because such questions have a high discrimination rate, they quickly sort providers into high- and low-competence groups. Taken together, these parameters describe the item characteristic curve for each of the items, which indicates, for every possible competence level, the likelihood that providers will complete the item.

To complete the estimation procedure, each provider was then assigned the corresponding competence score that best matches the exact response rate of questions they faced. For all providers, these scores are calculated using a maximum likelihood algorithm. In practice, this means that there is no other level of competence that is more consistent with their observed completion pattern across all history and examination items for all vignettes. For each provider, the assigned competence score indicates the likelihood they will complete each item. These scores are fully transitive across all providers regardless of differences between localized questionnaires, including whether or not each vignette or question was included in each study site.

References

1. Médecins Sans Frontières (MSF) International. Five things to know about our emergency paediatric project in Guinea-Bissau. Available at: https://www.msf.org/five-things-know-about-our-emergency-paediatric-project-guinea-bissau. *Online; Accessed 6 June* (2022).
2. World Health Organization Global Health Expenditure Database. Current health expenditure (% of GDP). Available at: https://data.worldbank.org/indicator/SH.XPD.CHEX.GD.ZS/. *Online; Accessed 25 July* (2023).
3. World Health Organization Global Health Expenditure Database. Current health expenditure per capita, PPP (current international $). Available at: https://data.worldbank.org/indicator/SH.XPD.CHEX.PP.CD/. *Online; Accessed 25 July* (2023).
4. Dossajee, H., Obonyo, N. & Ahmed, S. M. Career preferences of final year medical students at a medical school in Kenya–A cross sectional study. *BMC Med. Educ.* **16**, 1–10 (2016).
5. Muthaura, P. N., Khamis, T., Ahmed, M. & Hussain, S. R. Perceptions of the preparedness of medical graduates for internship responsibilities in district hospitals in Kenya: A qualitative study. *BMC Med. Educ.* **15**, 1–12 (2015).
6. Ramalanjaona, G. Emergency medicine in Madagascar. *Annals Emerg. Medicine* **31**, 766–768 (1998).
7. Sawatsky, A. P., Parekh, N., Muula, A. S. & Bui, T. Specialization training in Malawi: A qualitative study on the perspectives of medical students graduating from the University of Malawi College of Medicine. *BMC Med. Educ.* **14**, 1–10 (2014).
8. Muula, A. S. & Broadhead, R. L. South Africa's role in medical training in Malawi. *South Afr. Med. J.* **93**, 119–120 (2003).
9. WHO. World health organization - mozambique website (2022).
10. Bruce, J. C., Dippenaar, J., Schmollgruber, S., Mphuthi, D. D. & Huiskamp, A. Advancing nursing scholarship: The Mozambique model. *Glob. Heal. Action* **10**, 1351116 (2017).
11. Sherr, K. *et al.* Brain drain and health workforce distortions in Mozambique. *PloS One* **7**, e35840 (2012).
12. EduCeleb. Niger to get university of health and medical sciences. Available at: https://educeleb.com/niger-to-get-university-of-health-and-medical-sciences/. *Online; Accessed 6 June* (2022).
13. Oladipo, A. T. *et al.* Challenges with medical education in Nigeria in the COVID-19 era. *The Pan Afr. Med. J.* **37** (2020).
14. Federal Government of Nigeria. Second National Strategic Health Development Plan 2018-2022. (2018).
15. Médecins Sans Frontières (MSF) International. Training the future doctors of Sierra Leone while saving lives. Available at: https://www.msf-azg.be/en/

training-the-future-doctors-of-sierra-leone-while-saving-lives. *Online; Accessed 7 June* (2022).

16. Teclessou, J. N. *et al.* Factors influencing student's specialty choices in Lomé faculty of medicine (Togo). *BMC Med. Educ.* **21**, 1–8 (2021).

17. Kizito, S. *et al.* Career intentions of final year medical students in Uganda after graduating: The burden of brain drain. *BMC Med. Educ.* **15**, 1–7 (2015).

Figure 1. Provider vignette performance in country sub-populations

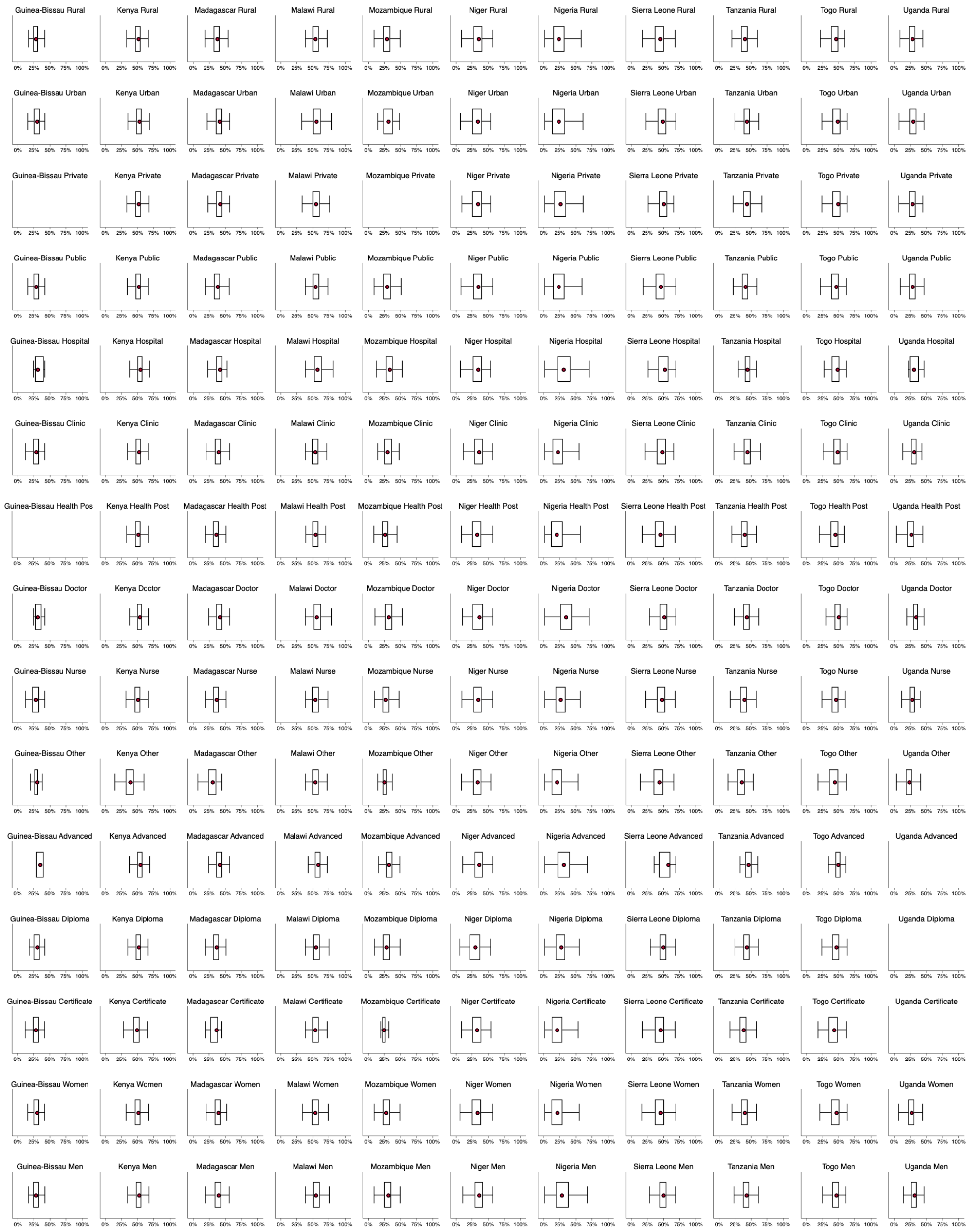

Note: This figure illustrates, each such sub-population, the distribution of the expected share of correctly managed vignettes for a provider at the median, outer Tukey adjacent values, and 25th and 75th percentiles (IQR) of the competence score distribution in that sub-population. Expected correct management is the individual provider's predicted proportion of correctly managed vignettes resulting from a logistic regression of correct management for each vignette on the competence score with country indicator controls.

Figure 2. Diagnostic performance is strongly unidimensional

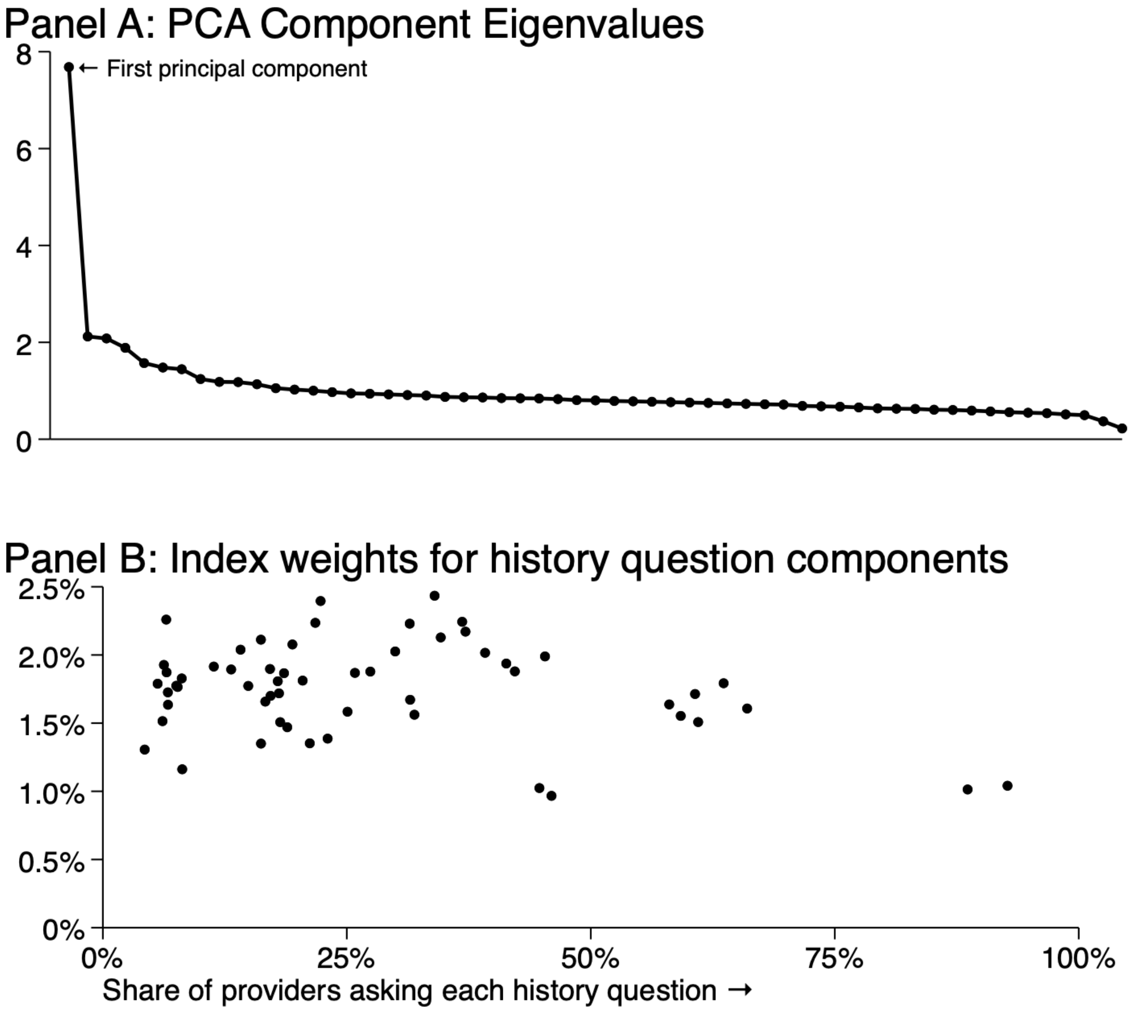

Note: This figure illustrates a validity check for unidimensionality in competence scores. All 57 vignette history questions that had over 10,000 observations were analyzed using principal components analysis (PCA). Panel A reports the ranked eigenvalues across the principal components determining the underlying variation; the first principal component has the highest eigenvalue. Panel B reports the relative contribution of each possible history question to the first principal component on the vertical axis, plotted against the share of providers completing that question on the horizontal axis. No question had negative contribution and there were no outliers with disproportionate contribution.

Figure 3. Demonstrative competence score components, with predicted and actual completion by provider score

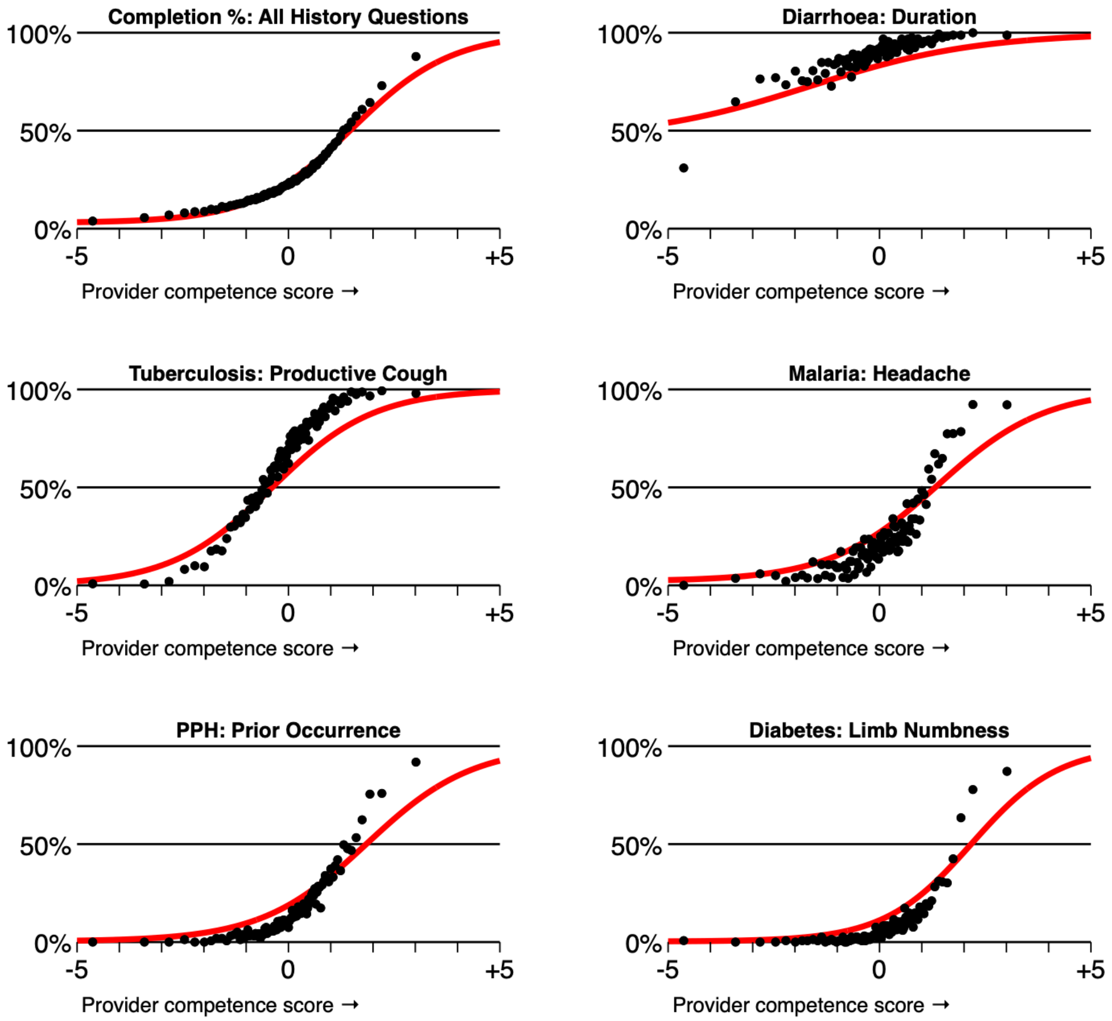

Note: This figure illustrates the predicted and actual performance from item response theory (IRT) competence scores (horizontal axis) and completion of all vignettes history questions as well as several illustrative examples of the questions. The vertical axis (0-100%) reports the completion rates for the various questions as well as the summary measure. The overlaid line represents theoretical predicted performance from the IRT estimation procedure; the dots are actual performance of providers in 100 bins corresponding to competence percentiles.

Figure 4. Discrimination and difficulty parameters for all IRT vignette diagnostic items

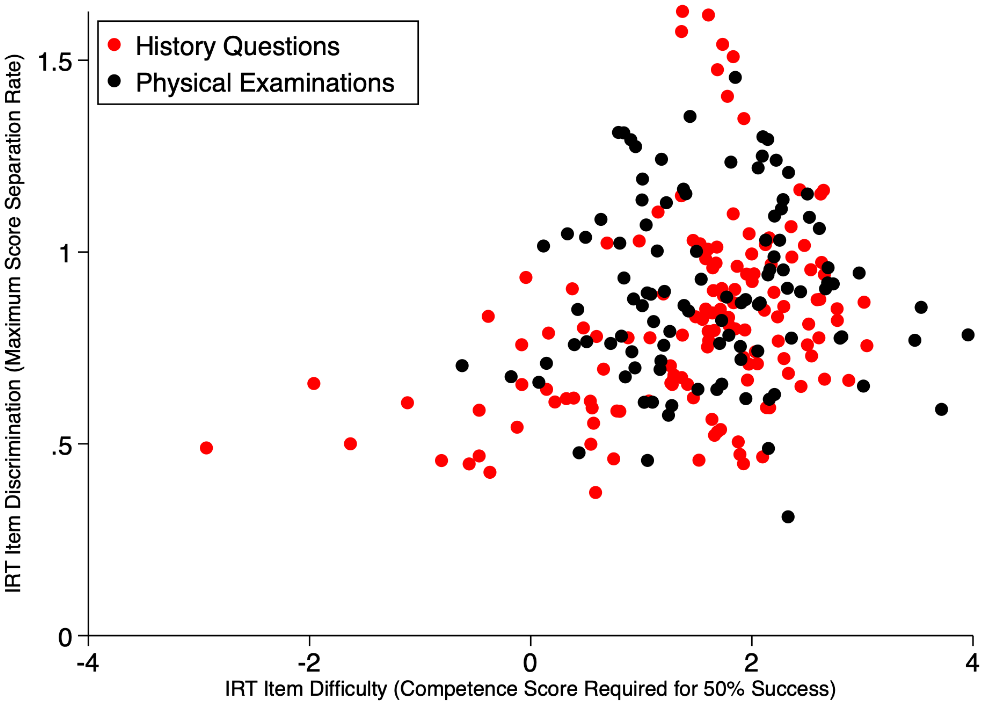

Note: This figure plots each of 241 history questions and physical examinations used in calculating IRT competence scores. The item difficulty is defined as the competence score required for a given provider to achieve an expected 50% success rate on the item, and the item discrimination is defined as the maximum slope of the item response curve.

Figure 5. Checklist completion for each condition is well-predicted by IRT scores

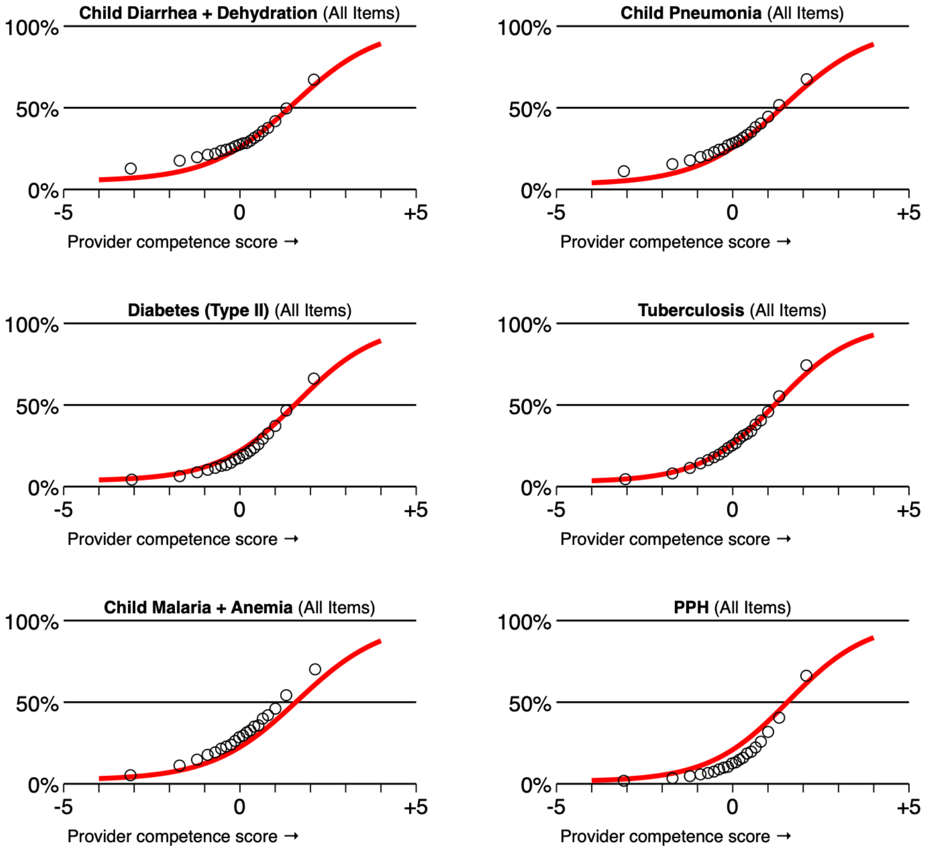

Note: This figure illustrates a validity check for fit of competence scores on completion of the diagnostic checklists for each vignette condition. This figure illustrates the relationship between the item response theory (IRT) competence scores (horizontal axis) and completion of all vignettes history questions for each vignette separately. The vertical axis (0-100%) reports the completion rates for the various questions as well as the summary measure. The overlaid line represents the theoretical relationship between the two; the dots are binned-scatterplots of the actual data with 20 quantiles.

Figure 6. Item characteristic curves for all IRT vignette diagnostic exams

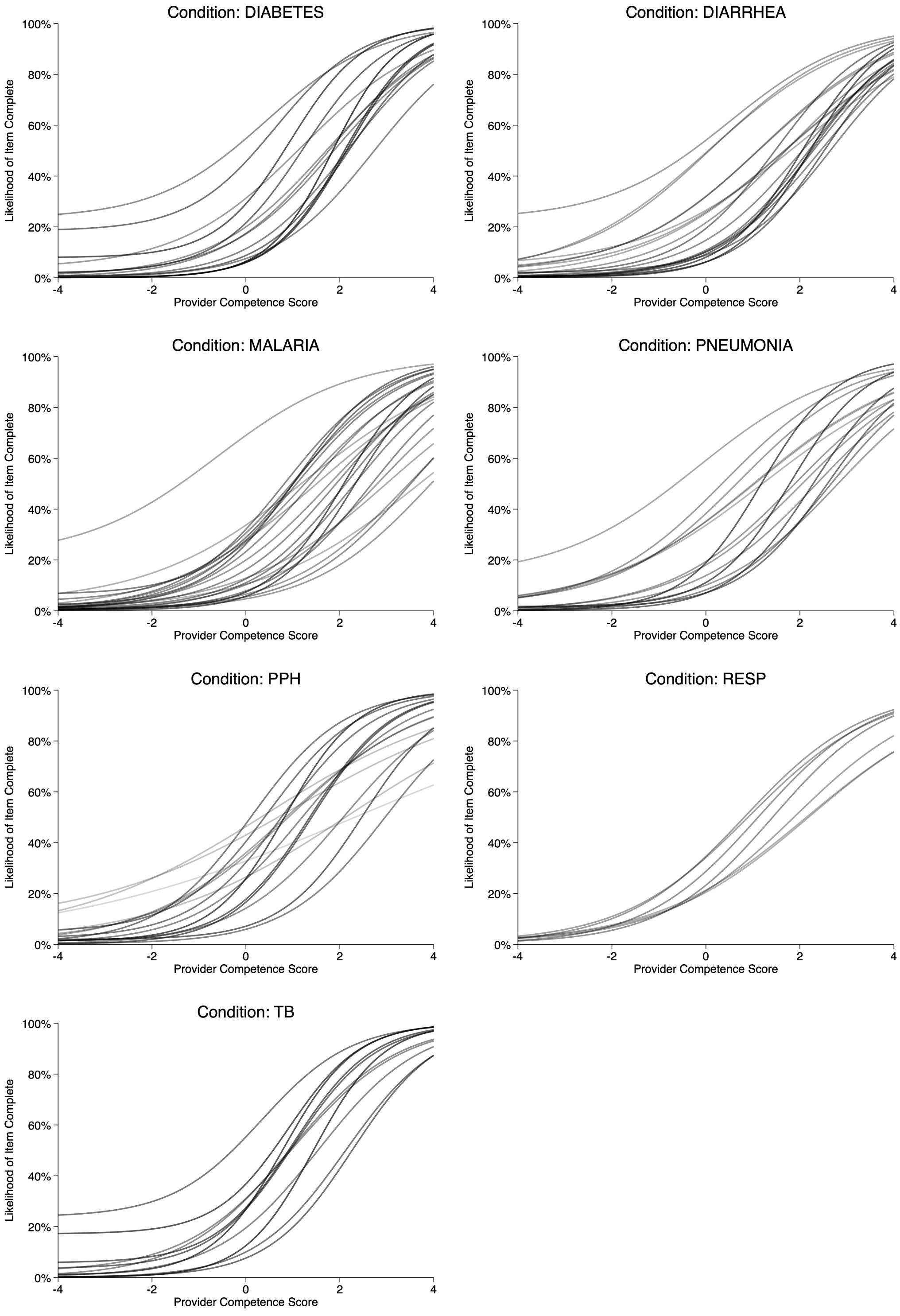

Note: This figure illustrates the item response curves for each of the diagnostic examinations in the seven vignette conditions. The curve plots the expected likelihood of success, defined as completion of the item in the vignette, for a provider with the competence score shown on the horizontal axis. Items with higher discrimination parameters are shown in darker fill.

Figure 7. Item characteristic curves for all IRT vignette diagnostic questions

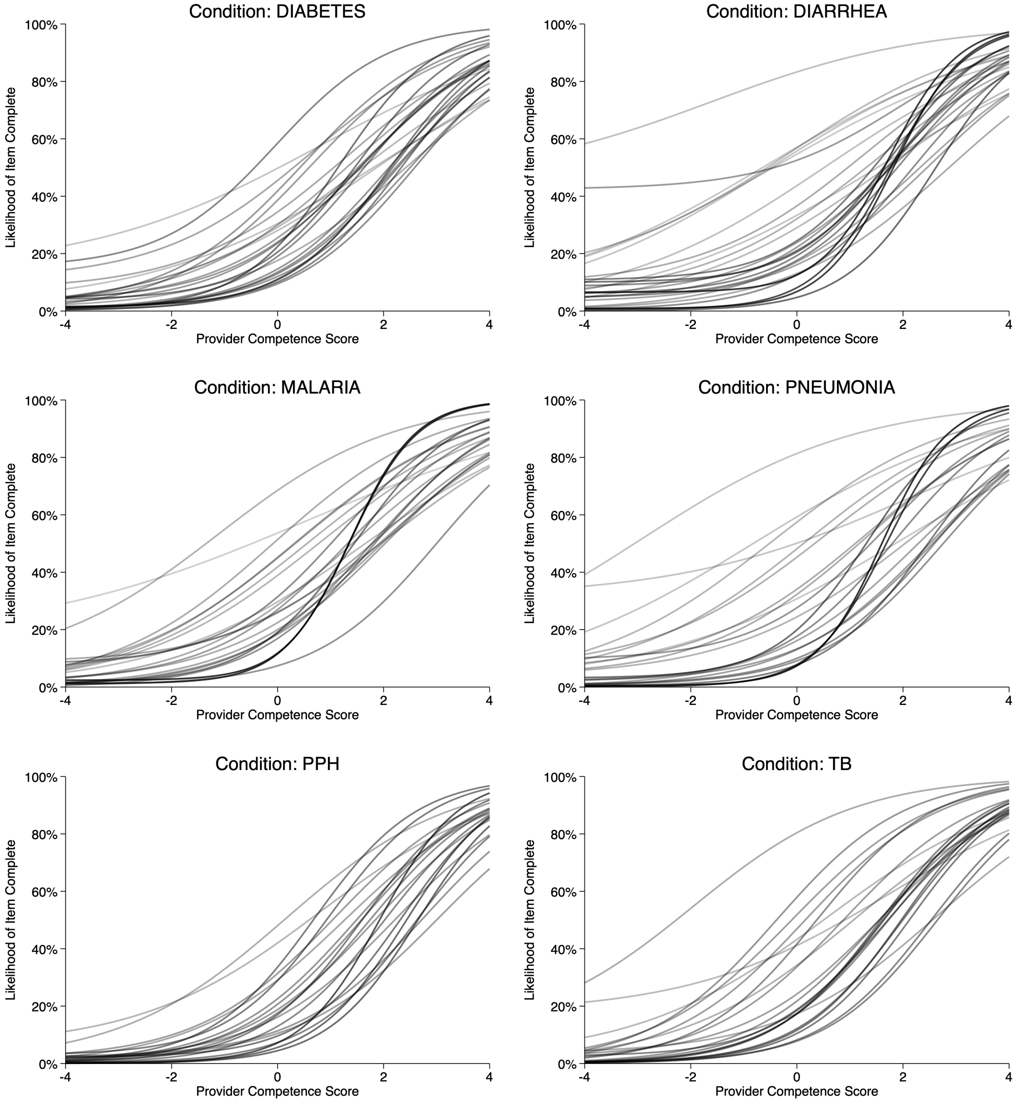

Note: This figure illustrates the item response curves for each of the diagnostic questions in the seven vignette conditions (RESP has none). The curve plots the expected likelihood of success, defined as completion of the item in the vignette, for a provider with the competence score shown on the horizontal axis. Items with higher discrimination parameters are shown in darker fill.

Table 1. Cadre classifications by professional title and country

(1)	(2)	(3)
**Doctor**	**Nurse**	**Other (Degree = Doctor, else Para-Professional)**
	**Guinea-Bissau**	
Director	Enfermeiro Geralista	Outra (especificar)
Director clínico	Parteira (com diploma)	
Médica de Saúde Pública	Auxiliar de Enfermagem	
Médico Especialista	Auxiliar de Parteira	
Médico Geralista		
	**Kenya**	
Physician/Medical Doctor (Specialist)	Nurse (Specialist)	Laboratory Technician/Technologist
Medical Officer	BSc. Nurse	Pharmacist/Pharmaceutical Technologist
Clinical Officer (Specialist)	KRCHN	Dentist
Clinical Officer	KECHN	Public Health Officer
		Health Administrative Officer
		Health Records Information Officer
		Nutritionist
		Medical Engineer
		Accounts Staff
		Procurement Staff
	**Madagascar**	
medecin specialiste	infirmier titulaire/sage femme titulaire	infirmier titulaire/sage femme titulaire
medecin generaliste	infirmier / sage femme	infirmier / sage femme
medecin du travail		auxiliaire medical/infirmier auxiliaire
medecin clinique		assistant maternite et pediatrie
		assistants medicaux zones rurales
	**Malawi**	
Physician/Medical Doctor (Specialist)	Registered Nurse Midwife	Medical Assistant
Physician/Medical Doctor(Generalist)	Community Health Nurse	Lab/Pharmacy
Medical Officer	Auxiliary nurse	Patient Aide
Clinical Officer	Nurse/Midwife Technician	Rural Medical Aides
	Community Midwife Assistant	Environmental health officer
	Nurse technician	Matron
		Health surveillance assistant
	**Mozambique**	
Director	Parteira (com diploma)	Assistente Técnico de Saúde
Director clínico	Parteira Auxiliar	Auxiliar Técnico de Saúde
Médica de Saúde Pública	Parteira Permanente	Cuidador
Médica Hospitalar		Técnico de laboratório
Médica Generalista		Especialista de Saúde
Técnico Superior de Saúde N1		Técnico de laboratório sénior
Técnico Superior de Saúde N2		Agente Polivalente Elementar
Técnico Especializado de Saúde		
Técnico de Saúde		
	**Niger**	
Médecin spécialiste	Technicien supérieur en soins infirmiers	Technicien supérieur en chirurgie
Médecin généraliste	Infirmier diplômée d'Etat	Technicien supérieur en anesthésie
	Sage-femme diplômée d'Etat	Techniciens de laboratoire
	Infirmier accoucheur	Techniciens d'assainissement et d'hygiène
	Infirmier certifié	Assistants sociaux
		Agent de santé de base
		Agent d'hygiène et d'assainissement
		Aide-assistant de l'action sociale
		Infirmier auxiliaire
	**Nigeria**	
Chief Medical Officer	Director of Nursing Services (DNS)	Community Health Officer
Specialist physician	Deputy DNS (DDNS)	Community Health Assistant
Medical Officer	Chief Nursing Officer (CNO)	Community Health Extension Worker (CHEW)
	Assistant Chief Nursing Officer (Asst CNO)	Junior CHEW
	Principal Nursing Officer I(PNO I)	Env Health Assistant
	Principal Nursing Officer II(PNO II)	Health Attendants/Auxiliary Nurses
	Senior Nursing Officer (SNO)	Laboratory Scientist
	Nursing Sister / Superintendent	Pharmacist
	Nursing Officer (NO)	Env Health Officer
	**Sierra Leone**	
Physician/Medical Doctor (Specialist)	Sierra Leone	Community Health Officer
Medical Officer	Physician/Medical Doctor (Specialist)	Community Health Assistant
House Officer	Medical Officer	Laboratory Technician
	House Officer	Maternal and Child Health (MCH) Aide
		Nursing Aide
		Nutritionist
		HIV Counsellors
		Anesthetist
		Physiotherapist
		Radiologist
		CHW
	**Tanzania**	
Physician/Medical Doctor (Specialist)	Nurse/Nurse Midwife	Lab/Pharmacy
Physician/Medical Doctor(Generalist)		Public Health Worker (Officer)
Medical Officer		Medical Attendant/ Nurse Assistant
Assistant Medical Officer		Maternity and Child Health (MCH) Aide
Clinical Officer		Rural Medical Aides
Assistant Clinical Officer		
	**Togo**	
Spécialiste	Infirmier diplômée d'Etat	Assistant permanent
Médecin généraliste	Infirmier auxiliaire d'Etat	Accoucheuse auxiliaire d'Etat
Assistant médical	Infirmier permanent	Accoucheuses permanente
		Aide-soignante
		Ingénieur des travaux biologiques
		Techniciens (supérieurs) de laboratoire
		Agents d'hygiène
	**Uganda**	
In charge	BSc Nurse	Enrolled Midwife
Specialist	Registered Nurse	Nurse Aide
Medical Officer	Enrolled Nurse	Public Health Officer (PHO)
Clinical Officer	Comprehensive Nurse	Dentist
	Registered Midwife	Pharmacist
		Laboratory Technician/ Technologist

Note: The SDI program categorized each provider as a “Doctor”, “Nurse”, or “Other”. This table reports each professional classification that was reported in each country during the roster recording and how they were mapped to the cadres.

Table 2. Medical vignette scenario descriptions

Medical Condition	(1) Opening Statement	(2) Correct Treatment
**Child Diarrhea + Severe Dehydration**	"I am a mother of a 13 month old boy. His name is Noel. My son has diarrhea."	IV fluid rehydration, nasogastric tube rehydration, or ORS plus zinc.
**Child Pneumonia**	"I am the mother of this 5 year old girl. Her name is Sia. She has a cough."	Amoxicillin, benzylpenicillin, or cotrimoxazole.
**Diabetes (Type II)**	"My name is Jack. I am worried that something is wrong with me. I feel weak and without energy even though I feel hungry often and eat frequently. I am 48 years old and work as a clerk."	Oral hypoglycemic or referral to a specialized clinic.
**Tuberculosis**	"My name is Bakari. I am 40 years old and I have been suffering from a fever and cough for some time."	Combination therapy (with or without correct dosage, drug names, and timing) or referral to TB clinic.
**Child Malaria + Anemia**	"I am the mother of this 4-year old boy. His name is Sangeti. He has had a fever for some time. Now he is worse, so I have come to you for help."	Artemether-lumefantrine (with or without correct dosage), artemisinin combination therapy (with or without correct dosage), or artesunate-amodiaquine. In addition to anti-malarials, the provider must to prescribe paracetamol and iron for the anemia.
**Post-Partum Hemorrhage**	"My name is Fatuma. I am 26 years old and I have vaginal bleeding 24 hours after delivery in a health facility."	Determine cause, IV line, uterine massage, and some type of uterotonic, prostaglandin or foley catheter must all be specified to be considered correct.
**Neonatal Asphyxia**	"A mother gives birth to a baby. The newborn is not crying. The newborn fails to establish regular breathing and appears pail and slightly blue. What do you do?"	Call for help, keep baby warm dry the baby, check if baby is breathing, place baby in a natural position, clear the airway, check heartrate and provide ventilation at least half must be taken to be considered correct.

Note: This table reports the vignette conditions that were conducted (which are also the correct response required for the diagnosis), the representative initial statement that the enumerator used to introduce their complaint (names are localized), and the SDI program's internationally-harmonized standard of correct case management.

Table 3. Range of correct vignette management in each country sub-population

	Guinea-Bissau	Kenya	Madagascar	Malawi	Mozambique	Niger	Nigeria	Sierra Leone	Tanzania	Togo	Uganda
**Observations**	103	4485	619	1519	683	594	5017	829	1018	527	733
**Mean Age**	38.0	36.9	43.5	36.9	32.7	35.0	40.6	41.1	42.5	38.1	34.5
**Rural**	**28.3%**	**49.7%**	**35.9%**	**54.5%**	**28.6%**	**33.8%**	**24.1%**	**42.9%**	**39.2%**	**42.9%**	**27.1%**
IQR 25th	24.2%	45.8%	31.9%	49.3%	23.3%	27.2%	14.7%	36.5%	34.3%	37.9%	22.5%
IQR 75th	31.3%	54.3%	41.3%	58.7%	34.0%	40.8%	32.4%	50.1%	44.6%	49.2%	32.7%
**Urban**	**29.3%**	**51.6%**	**39.4%**	**56.5%**	**30.9%**	**32.7%**	**23.8%**	**46.7%**	**43.4%**	**46.1%**	**28.3%**
IQR 25th	25.1%	47.5%	35.4%	49.7%	23.7%	25.2%	12.7%	41.1%	38.5%	40.1%	23.2%
IQR 75th	34.1%	55.9%	44.8%	61.6%	37.9%	39.8%	33.2%	54.0%	47.9%	52.5%	34.4%
**Private**		**50.3%**	**40.2%**	**55.4%**		**32.2%**	**25.4%**	**48.1%**	**43.1%**	**45.7%**	**27.4%**
IQR 25th		46.1%	36.0%	49.3%		25.0%	15.5%	43.3%	37.6%	39.9%	22.5%
IQR 75th		54.8%	45.0%	60.1%		40.0%	34.9%	55.5%	49.1%	52.4%	33.1%
**Public**	**28.7%**	**50.2%**	**36.6%**	**55.1%**	**29.0%**	**33.4%**	**24.1%**	**44.0%**	**40.1%**	**43.7%**	**27.3%**
IQR 25th	24.8%	46.4%	32.5%	49.4%	23.4%	26.8%	14.3%	37.9%	35.7%	38.2%	22.8%
IQR 75th	33.1%	54.6%	41.9%	59.3%	34.8%	40.2%	32.5%	51.2%	45.0%	49.8%	33.2%
**Hospital**	**33.0%**	**52.8%**	**40.7%**	**58.4%**	**32.5%**	**33.0%**	**31.2%**	**49.4%**	**44.6%**	**45.1%**	**31.6%**
IQR 25th	27.2%	49.3%	36.0%	51.2%	27.2%	26.0%	21.2%	42.2%	40.3%	38.9%	23.5%
IQR 75th	40.2%	57.1%	45.2%	63.4%	37.8%	40.2%	41.8%	57.5%	48.5%	50.8%	38.3%
**Clinic**	**28.5%**	**50.6%**	**38.1%**	**54.1%**	**30.5%**	**34.5%**	**22.4%**	**45.8%**	**44.1%**	**46.2%**	**29.9%**
IQR 25th	24.2%	46.6%	33.9%	48.6%	24.9%	28.5%	13.6%	39.7%	38.5%	41.7%	25.9%
IQR 75th	32.8%	54.7%	43.4%	57.9%	36.5%	41.5%	30.1%	53.4%	49.2%	52.4%	34.5%
**Health Post**		**49.6%**	**34.7%**	**54.0%**	**26.3%**	**32.2%**	**21.2%**	**43.2%**	**38.9%**	**42.3%**	**25.1%**
IQR 25th		45.7%	31.2%	49.3%	21.9%	25.0%	11.3%	37.1%	34.2%	37.1%	19.7%
IQR 75th		54.2%	40.1%	57.9%	31.0%	39.2%	29.7%	50.4%	44.3%	49.2%	31.0%
**Doctor**	**31.6%**	**52.4%**	**40.0%**	**57.1%**	**31.1%**	**34.2%**	**34.4%**	**49.7%**	**42.8%**	**47.5%**	**32.5%**
IQR 25th	27.2%	48.8%	36.1%	50.7%	25.9%	25.7%	25.9%	44.0%	37.7%	43.4%	29.6%
IQR 75th	36.8%	56.2%	44.9%	62.1%	36.8%	41.7%	44.1%	55.5%	47.5%	52.5%	37.0%
**Nurse**	**28.1%**	**49.0%**	**35.2%**	**54.0%**	**27.1%**	**33.1%**	**26.9%**	**45.8%**	**38.0%**	**43.9%**	**27.1%**
IQR 25th	22.7%	45.0%	31.2%	48.5%	22.3%	27.2%	18.4%	39.9%	32.8%	38.9%	23.5%
IQR 75th	33.0%	53.7%	40.1%	58.7%	32.5%	39.7%	34.1%	52.3%	43.2%	49.3%	32.0%
**Other**	**28.5%**	**36.8%**	**28.8%**	**54.5%**	**25.9%**	**32.5%**	**21.2%**	**41.6%**	**33.8%**	**41.7%**	**22.2%**
IQR 25th	26.0%	31.6%	23.4%	49.1%	22.6%	26.6%	12.1%	34.7%	28.1%	34.7%	17.7%
IQR 75th	31.2%	43.7%	36.1%	58.5%	28.4%	39.4%	28.7%	49.3%	40.0%	49.1%	27.8%
**Advanced**	**34.4%**	**52.7%**	**40.0%**	**58.9%**	**32.0%**	**34.7%**	**31.5%**	**52.7%**	**45.9%**	**48.0%**	
IQR 25th	28.6%	49.1%	36.1%	52.5%	27.7%	28.9%	21.5%	43.2%	40.7%	44.8%	
IQR 75th	40.2%	57.2%	44.9%	61.2%	37.3%	41.4%	41.0%	60.4%	50.5%	52.4%	
**Diploma**	**29.5%**	**50.6%**	**35.3%**	**55.9%**	**28.0%**	**29.4%**	**26.5%**	**48.7%**	**42.8%**	**44.6%**	
IQR 25th	25.0%	46.8%	31.2%	49.9%	22.8%	20.7%	18.5%	44.2%	37.9%	39.5%	
IQR 75th	34.0%	54.7%	40.3%	60.1%	33.4%	37.6%	33.4%	54.0%	47.4%	50.5%	
**Certificate**	**28.4%**	**46.9%**	**32.6%**	**54.6%**	**24.1%**	**32.1%**	**21.3%**	**43.2%**	**36.9%**	**40.8%**	
IQR 25th	24.1%	42.8%	27.0%	49.0%	22.2%	25.4%	12.2%	36.9%	32.2%	34.1%	
IQR 75th	32.8%	52.8%	38.9%	58.6%	26.9%	39.3%	28.9%	50.6%	42.5%	48.3%	
**Women**	**28.9%**	**49.5%**	**37.8%**	**54.5%**	**28.1%**	**32.2%**	**21.4%**	**43.2%**	**39.0%**	**44.1%**	**25.8%**
IQR 25th	24.9%	45.3%	33.7%	48.3%	22.7%	24.5%	12.0%	36.8%	34.3%	37.9%	21.3%
IQR 75th	33.1%	54.1%	42.7%	58.7%	33.4%	38.9%	29.2%	50.6%	44.5%	50.8%	31.1%
**Men**	**28.6%**	**51.0%**	**37.9%**	**55.7%**	**30.0%**	**34.7%**	**28.9%**	**48.5%**	**42.3%**	**44.1%**	**29.8%**
IQR 25th	24.1%	47.1%	33.6%	49.8%	24.6%	29.0%	18.6%	43.8%	37.3%	39.7%	25.9%
IQR 75th	32.6%	55.4%	43.6%	60.2%	35.4%	41.6%	39.0%	54.3%	46.9%	50.2%	35.0%

Note: This table reports the mean expected number of correctly managed vignettes (bold) of providers in each sub-population within each country. For each such sub-population, it then reports the expected share of correctly managed vignettes for a provider at the 25th and 75th percentile of the competence score distribution in that sub-population. Expected correct management is the individual provider's predicted proportion of correctly managed vignettes resulting from a logistic regression of correct management for each vignette on the competence score with country indicator controls.

Table 4. Item characteristics for all IRT vignette diagnostic exams

Vignette	Item	Difficulty	Discrimination	Guess Rate
DIABETES	History: Duration of symptoms	-0.04	0.93	15.2%
DIABETES	Physical Exam: Blood pressure	0.43	0.85	23.2%
DIABETES	History: Urinary output	0.48	0.80	2.2%
DIABETES	History: Thirst	0.60	0.78	0.0%
DIABETES	Physical Exam: Temperature	0.64	1.09	18.4%
DIABETES	History: Appetite	0.66	0.69	11.0%
DIABETES	History: Fever	0.75	0.46	14.2%
DIABETES	Physical Exam: Pulse	0.95	1.27	7.9%
DIABETES	History: Diabetes in family	1.15	1.10	0.8%
DIABETES	Physical Exam: Weight	1.20	0.76	3.7%
DIABETES	Physical Exam: Respiratory rate	1.23	1.13	1.9%
DIABETES	History: Medication/treatment	1.30	0.68	7.4%
DIABETES	History: Headache	1.37	0.67	2.1%
DIABETES	History: Dizziness or fainting	1.53	1.02	3.9%
DIABETES	History: Do you have other symptoms?	1.60	0.75	3.2%
DIABETES	History: Did you eat this morning?	1.60	0.79	2.2%
DIABETES	History: Vomiting	1.64	0.56	1.1%
DIABETES	History: Hypertension	1.65	0.90	0.7%
DIABETES	History: Previous health checks	1.66	0.80	3.8%
DIABETES	Physical Exam: Abdomen/liver	1.73	0.82	0.7%
DIABETES	Physical Exam: Auscultate chest	1.77	0.88	0.2%
DIABETES	Physical Exam: Lungs	1.85	1.46	0.1%
DIABETES	History: Cough	1.88	0.51	0.2%
DIABETES	History: Diarrhea	1.89	0.47	2.0%
DIABETES	Physical Exam: Sunken eyes	1.90	0.87	1.4%
DIABETES	Physical Exam: Neurological exam, lower extremities	2.10	1.30	0.0%
DIABETES	History: Difficulty breathing	2.11	0.85	0.5%
DIABETES	Physical Exam: Neurological exam, upper extremities	2.14	1.29	0.1%
DIABETES	History: Ask about eye sight	2.16	1.04	1.4%
DIABETES	Physical Exam: Height	2.16	0.95	0.2%
DIABETES	History: Lower limb numbness/excessively cold or hot feeling in the feet	2.17	0.97	0.3%
DIABETES	Physical Exam: Oral examination	2.20	1.09	0.6%
DIABETES	History: Smoking	2.23	0.83	0.1%
DIABETES	History: TB/HIV in family	2.29	0.86	0.8%
DIABETES	History: Exercise	2.36	0.99	1.4%
DIABETES	History: Backache or joint paints	2.44	0.65	0.8%
DIABETES	History: Convulsions	2.51	0.81	0.6%
DIABETES	History: Sunken eyes	2.59	0.88	0.1%
DIABETES	Physical Exam: Fundoscopy	2.74	0.92	0.0%

Vignette	Item	Difficulty	Discrimination	Guess Rate
DIARRHEA	History: Duration of diarrhea	-1.63	0.50	45.5%
DIARRHEA	History: Vomiting	-0.56	0.45	2.0%
DIARRHEA	History: Consistency of stool	-0.47	0.47	0.7%
DIARRHEA	History: Fever	-0.37	0.43	1.6%
DIARRHEA	History: Frequency of diarrhea	-0.12	0.54	10.6%
DIARRHEA	Physical Exam: Skin pinch	0.07	0.66	0.8%
DIARRHEA	Physical Exam: Sunken eyes	0.14	0.71	2.5%
DIARRHEA	Physical Exam: Temperature	0.51	0.77	22.9%
DIARRHEA	History: Blood in stool	0.54	0.50	0.7%
DIARRHEA	Physical Exam: Weight	1.17	0.69	1.9%
DIARRHEA	Physical Exam: General health condition (awake/lethargic/tiredness/fatigue)	1.18	0.72	2.6%
DIARRHEA	History: Breastfeeding	1.27	0.66	9.1%
DIARRHEA	History: Does he have other symptoms?	1.47	0.62	4.9%
DIARRHEA	Physical Exam: Respiratory rate	1.50	1.00	1.5%
DIARRHEA	History: Breastfeeding well	1.52	0.46	0.1%
DIARRHEA	History: Food other than breastmilk	1.68	1.01	10.8%
DIARRHEA	Physical Exam: Offer drink	1.68	0.64	0.0%
DIARRHEA	History: Cough	1.69	0.53	0.1%
DIARRHEA	Physical Exam: Mucour membrane (mouth)	1.71	0.76	0.3%
DIARRHEA	Physical Exam: Look for palmar pallor (or other signs of anaemia)	1.73	0.66	2.0%
DIARRHEA	History: General condition	1.74	1.54	6.6%
DIARRHEA	History: HIV sero-status of child	1.78	1.41	0.6%
DIARRHEA	History: Vaccination status	1.79	0.81	4.0%
DIARRHEA	History: If yes, how often	1.83	1.51	0.9%
DIARRHEA	History: What else do you feed him?	1.83	1.10	10.0%
DIARRHEA	History: Hand washing practice	1.87	0.96	5.9%
DIARRHEA	History: Abdominal discomfort/cramps	1.92	0.73	5.0%
DIARRHEA	History: Medication/treatment	1.94	0.80	42.4%
DIARRHEA	Physical Exam: Agitated/irritable	1.95	0.62	4.4%
DIARRHEA	History: How is the food given?	1.95	0.94	8.6%
DIARRHEA	History: Who prepares the food?	2.00	0.92	4.7%
DIARRHEA	Physical Exam: Check for visible severe wasting	2.06	1.22	0.6%
DIARRHEA	Physical Exam: Palpate spleen	2.06	0.86	0.4%
DIARRHEA	History: Other family members/neighbours have diarrhea	2.20	0.89	0.4%
DIARRHEA	Physical Exam: Check weight (against a growth chart)	2.20	0.99	0.7%
DIARRHEA	Physical Exam: Blood pressure	2.22	1.24	0.2%
DIARRHEA	Physical Exam: Pulse/heart rate	2.25	1.03	0.4%
DIARRHEA	Physical Exam: Look for oedema of both feet (swollen feet)	2.28	0.95	0.1%
DIARRHEA	History: Convulsions	2.33	0.68	0.3%
DIARRHEA	Physical Exam: Check ear/throat	2.44	0.90	0.1%

DIARRHEA	Physical Exam: Neck stiffness	2.52	1.09	0.2%
DIARRHEA	History: Last deworming	2.54	0.73	3.0%
DIARRHEA	History: Measles now or in past 3 mon?	2.62	1.15	0.1%
DIARRHEA	Physical Exam: Height/length	2.69	0.96	2.0%
DIARRHEA	History: Tears	2.88	0.67	0.2%

Vignette	Item	Difficulty	Discrimination	Guess Rate
MALARIA	History: Duration of fever	-1.11	0.61	6.6%
MALARIA	Physical Exam: Temperature	-0.62	0.70	21.1%
MALARIA	History: Vomiting	-0.08	0.65	0.2%
MALARIA	History: Appetite	0.32	0.62	0.3%
MALARIA	History: Cough	0.39	0.62	1.7%
MALARIA	History: Diarrhoea	0.56	0.59	0.0%
MALARIA	History: Medication/treatment	0.59	0.37	16.5%
MALARIA	History: Pattern/history of fever	0.78	0.59	0.2%
MALARIA	Physical Exam: Eyes, pale colour?	0.84	0.93	1.2%
MALARIA	Physical Exam: Respiratory rate	1.04	1.07	2.2%
MALARIA	Physical Exam: Weight	1.05	0.89	3.5%
MALARIA	Physical Exam: Hands (palmar pallor)	1.09	0.89	0.4%
MALARIA	Physical Exam: Pulse	1.14	1.00	6.3%
MALARIA	History: Other symptoms?	1.20	0.89	7.9%
MALARIA	Physical Exam: Tongue	1.26	0.79	0.2%
MALARIA	Physical Exam: Yellow eyes/jaundice	1.28	0.60	2.9%
MALARIA	History: HIV status of mother	1.36	1.57	1.3%
MALARIA	History: Headache	1.37	0.78	2.1%
MALARIA	History: HIV status of child	1.37	1.63	2.4%
MALARIA	Physical Exam: Responsiveness/general condition	1.39	0.86	0.0%
MALARIA	History: Shiver or sweat	1.42	0.66	0.4%
MALARIA	History: Lethargic or unconscious	1.47	1.03	1.3%
MALARIA	Physical Exam: Eyes, sunken?	1.51	0.64	0.2%
MALARIA	History: Convulsions	1.66	0.52	0.2%
MALARIA	History: Difficulty breathing	1.79	0.83	0.5%
MALARIA	Physical Exam: Abdomen/liver	1.79	0.78	0.3%
MALARIA	History: Vaccinations	1.83	0.87	0.0%
MALARIA	Physical Exam: Skin condition	1.94	0.88	1.0%
MALARIA	History: Urination	1.96	0.67	1.4%
MALARIA	History: Severity of cough	1.97	0.71	0.2%
MALARIA	History: Type of cough (productive or dry)	2.03	0.74	0.2%
MALARIA	History: Amount	2.05	0.71	8.6%
MALARIA	Physical Exam: Blood pressure	2.09	1.25	0.7%
MALARIA	History: Does child sleep under impregnated bed net?	2.13	0.59	5.4%
MALARIA	Physical Exam: Nail beds	2.15	0.94	1.0%
MALARIA	Physical Exam: Swelling of the feet	2.32	0.91	0.2%
MALARIA	Physical Exam: Height	2.33	1.21	0.1%
MALARIA	Physical Exam: Puffy face	2.69	0.92	0.1%
MALARIA	Physical Exam: Neck stiffness	2.80	0.78	0.0%
MALARIA	Physical Exam: Color of urine	3.01	0.65	0.6%
MALARIA	History: Measles now or in past 3 months	3.01	0.87	0.7%
MALARIA	Physical Exam: Red eyes	3.47	0.77	0.1%
MALARIA	Physical Exam: Skin rash	3.53	0.86	0.9%
MALARIA	Physical Exam: Runny nose	3.72	0.59	0.5%

MALARIA	Physical Exam: Koplik sign	3.96	0.78	0.4%

Vignette	Item	Difficulty	Discrimination	Guess Rate
PNEUMONIA	History: Duration of cough	-2.93	0.49	3.0%
PNEUMONIA	History: Fever	-0.81	0.46	0.4%
PNEUMONIA	History: Sputum production or dry cough	-0.47	0.59	1.5%
PNEUMONIA	Physical Exam: Temperature	-0.18	0.68	13.2%
PNEUMONIA	History: Chest pain/difficulty breathing	0.22	0.61	2.8%
PNEUMONIA	Physical Exam: Auscultate the chest	0.39	0.76	1.9%
PNEUMONIA	History: Blood/color of sputum	0.54	0.61	5.9%
PNEUMONIA	Physical Exam: Respiratory rate	0.72	0.76	2.7%
PNEUMONIA	Physical Exam: Weight	1.03	0.61	0.6%
PNEUMONIA	Physical Exam: Wheezing?	1.10	0.61	1.8%
PNEUMONIA	Physical Exam: General condition	1.18	1.24	0.2%
PNEUMONIA	Physical Exam: Observe breathing for lower chest wall indra	1.25	0.57	1.2%
PNEUMONIA	History: Vomiting	1.28	0.65	5.6%
PNEUMONIA	History: Appetite	1.29	0.67	3.7%
PNEUMONIA	History: Able to drink?	1.36	1.15	3.1%
PNEUMONIA	History: HIV status of child	1.61	1.62	0.5%
PNEUMONIA	History: Do you have other symptoms?	1.64	0.84	9.4%
PNEUMONIA	History: HIV status of mother	1.69	1.48	0.2%
PNEUMONIA	Physical Exam: Stridor	1.81	1.23	1.7%
PNEUMONIA	History: General condition (awake/lethargic)	1.85	0.90	2.1%
PNEUMONIA	Physical Exam: Observe for nasal flaring	1.90	0.75	0.1%
PNEUMONIA	History: Running nose	1.92	0.45	1.5%
PNEUMONIA	Physical Exam: Pulse/heart rate	2.05	0.74	0.2%
PNEUMONIA	History: Medication/treatment	2.10	0.47	31.3%
PNEUMONIA	History: Diarrhea	2.16	0.59	3.5%
PNEUMONIA	Physical Exam: Blood pressure	2.28	1.14	0.1%
PNEUMONIA	History: Family history of asthma	2.29	0.72	0.1%
PNEUMONIA	Physical Exam: Examine throat	2.36	0.78	0.0%
PNEUMONIA	History: Ear discharge	2.47	1.02	0.3%
PNEUMONIA	History: Convulsions	2.50	0.76	0.0%
PNEUMONIA	History: Redness of the eyes	2.61	0.78	1.9%
PNEUMONIA	Physical Exam: Height	2.61	1.06	1.3%
PNEUMONIA	History: Difficulty swallowing	2.61	0.88	0.9%
PNEUMONIA	Physical Exam: Lymph nodes	2.67	0.90	0.0%
PNEUMONIA	History: Recent history of measles	2.77	0.85	0.6%
PNEUMONIA	Physical Exam: Ears	2.81	0.78	0.1%

Vignette	Item	Difficulty	Discrimination	Guess Rate
PPH	Physical Exam: Pallor	0.12	1.02	0.7%
PPH	History: Amount	0.14	0.64	0.7%
PPH	Physical Exam: Laceration/tears of genital tract	0.44	0.48	2.8%
PPH	Physical Exam: Blood pressure	0.50	1.04	4.9%
PPH	History: Type of delivery	0.69	1.02	0.9%
PPH	Physical Exam: Temperature	0.81	1.02	2.5%
PPH	History: Placenta delivery	0.81	0.58	5.8%
PPH	Physical Exam: Pulse	0.84	1.31	1.3%
PPH	Physical Exam: Uterine palpation	0.86	0.67	0.1%
PPH	Physical Exam: Genital exam	0.94	0.70	1.2%
PPH	History: Gestity	0.98	1.03	3.0%
PPH	Physical Exam: Retained placenta	1.06	0.46	7.8%
PPH	History: Color of blood	1.08	0.78	1.7%
PPH	Physical Exam: Conjonctiva	1.21	0.90	0.5%
PPH	History: Pads	1.26	0.70	0.0%
PPH	Physical Exam: General condition	1.38	1.16	1.3%
PPH	Physical Exam: Respiratory rate	1.40	1.15	0.1%
PPH	History: Parity	1.49	0.83	0.2%
PPH	History: Duration of labor	1.58	0.85	0.0%
PPH	History: Attendance of ANC	1.60	1.01	0.6%
PPH	History: Do you have other symptoms?	1.74	0.89	0.1%
PPH	History: Bleeding in prior pregnancies	1.85	0.80	0.3%
PPH	History: Care immediately prior to delivery	1.93	1.35	0.3%
PPH	History: Hypertension	2.02	0.94	0.0%
PPH	Physical Exam: Weight	2.08	0.87	0.1%
PPH	Physical Exam: Ruptured uterus	2.15	0.49	0.7%
PPH	History: Given labor augmenting drugs during delivery	2.24	0.77	1.2%
PPH	Physical Exam: Cervix	2.33	0.31	0.1%
PPH	History: History of bleeding from cuts	2.35	1.07	2.3%
PPH	History: Multiple (twin) pregnancy	2.43	1.16	0.1%
PPH	Physical Exam: Capillary refill	2.50	1.15	2.0%
PPH	History: History of prolonged menses (fibroids)	2.63	0.97	0.2%
PPH	History: Excessive (amniotic) fluid during pregnancy	2.65	1.16	0.1%
PPH	History: Placenta praevia/abruption	2.77	0.82	2.3%
PPH	Physical Exam: Height	2.97	0.95	0.3%
PPH	History: Fever during delivery	3.04	0.76	1.5%

Vignette	Item	Difficulty	Discrimination	Guess Rate
RESP	Physical Exam: Observe respiration effort	0.82	0.78	0.2%
RESP	Physical Exam: Check heart rate	0.91	0.74	0.7%
RESP	Physical Exam: Look at neonate's color	1.11	0.82	0.0%
RESP	Physical Exam: Score neonate using AGPAR scale	1.43	0.85	0.4%
RESP	Physical Exam: Muscle tone	1.90	0.72	1.0%
RESP	Physical Exam: Test reflex irritability	2.16	0.62	0.5%
RESP	Physical Exam: APGAR score 0-4, <4	2.20	0.63	0.6%

Vignette	Item	Difficulty	Discrimination	Guess Rate
TB	History: Duration of cough	-1.96	0.66	9.3%
TB	History: Productive cough	-0.38	0.83	0.0%
TB	History: Blood in sputum	-0.08	0.76	0.6%
TB	History: (Night) sweats	0.16	0.79	0.1%
TB	Physical Exam: Take temperature	0.33	1.05	23.7%
TB	History: Weight loss	0.38	0.90	0.1%
TB	History: Presence of fever and pattern	0.57	0.55	1.8%
TB	Physical Exam: General condition	0.79	1.31	1.0%
TB	History: Chest pain/difficulty breathing	0.88	0.78	0.2%
TB	Physical Exam: Blood pressure	0.90	1.29	17.2%
TB	Physical Exam: Chest examination (auscultation or other)	0.93	0.88	0.2%
TB	Physical Exam: Take respiratory rate	1.01	1.14	3.5%
TB	Physical Exam: Check weight	1.01	0.86	2.2%
TB	Physical Exam: Take pulse rate	1.01	1.19	5.8%
TB	History: Appetite	1.07	0.61	0.1%
TB	Physical Exam: Check other systems	1.44	1.35	0.1%
TB	Physical Exam: Shortness of breath	1.54	0.93	0.3%
TB	History: Has this type of cough happened to others in family/dwelling?	1.55	0.82	0.0%
TB	History: TB in household	1.58	0.98	0.0%
TB	History: Do you have other symptoms?	1.61	0.77	2.3%
TB	History: History of contact with adult with prolonged cough in household	1.65	0.96	0.3%
TB	History: General health condition (tiredness/fatique)	1.68	0.97	2.5%
TB	History: HIV test taken	1.71	0.85	0.0%
TB	History: Have you been given medication/treatment for the cough/fever?	1.72	0.54	17.7%
TB	History: Do you smoke	1.73	0.90	0.0%
TB	History: Do you drink/take alcohol	1.98	1.05	0.4%
TB	History: Has this happened before?	2.00	1.00	0.2%
TB	History: Profession	2.12	1.02	0.2%
TB	Physical Exam: Retraction or decreased movement	2.13	1.03	0.0%
TB	Physical Exam: Check height	2.27	1.11	0.2%
TB	History: Normal diet	2.53	0.95	0.0%
TB	History: Have you indulged in high-risk sexual behavior	2.66	0.94	0.3%
TB	History: Treated for TB before?	2.66	0.67	3.3%
